# Supplementary material for: Enteral Feeding Interventions in the Prevention of Necrotizing Enterocolitis: A Systematic Review of Experimental and Clinical Studies
Source: Nutrients. 2021 May 19;13(5):1726. doi: 10.3390/nu13051726 (PMC8161173; doi:10.3390/nu13051726)
Supplement: Supplementary file 1 [file nutrients-13-01726-s001.zip › nutrients-1212808-supplementary.pdf]

Index supplementary files

Supplementary table S1. Pubmed search `page 2`

Supplementary table S2. Embase search page 6

Supplementary table S3. Cochrane library search page 9

Supplementary table S4. Overview of RCTs that were not incorporated in one of the included meta-analyses and had a too small sample size (<50% of infants in the meta-analysis on the same intervention) to be included as separate trial. page 15

Supplementary table S5. Overview included experimental animal studies on enteral feeding interventions for prevention of NEC page 17

Supplementary table S6. Overview included clinical trials on enteral feeding interventions for prevention of NEC in human infants page 61

Supplementary table S7. Overview included systematic reviews and meta-analysis on enteral feeding interventions for prevention of NEC in human infants page 72

Supplementary table S8. Risk of bias assessment of the included experimental animal studies (SYRCLE's risk of bias tool) page 76

Supplementary table S9. Risk of bias assessment of the included RCTs (Jadad scoring system) page 77

Supplementary table S10. Risk of bias assessment of the included meta-analyses (AMSTAR measurement tool) page 77

Supplementary table S11. GRADE approach scoring of clinical studies page 78

Supplementary table S1. Pubmed search

Search title	Search terms used in Pubmed
general feeding interventions	((("feeding"[All Fields] OR "feedings"[All Fields] OR "feeds"[All Fields]) AND ("intervention s"[All Fields] OR "interventions"[All Fields] OR "interventive"[All Fields] OR "methods"[MeSH Terms] OR "methods"[All Fields] OR "intervention"[All Fields] OR "interventional"[All Fields])) OR (("nutrition s"[All Fields] OR "nutritional status"[MeSH Terms] OR ("nutritional"[All Fields] AND "status"[All Fields]) OR "nutritional status"[All Fields] OR "nutrition"[All Fields] OR "nutritional sciences"[MeSH Terms] OR ("nutritional"[All Fields] AND "sciences"[All Fields]) OR "nutritional sciences"[All Fields] OR "nutritional"[All Fields] OR "nutritionals"[All Fields] OR "nutritions"[All Fields] OR "nutritive"[All Fields]) AND ("intervention s"[All Fields] OR "interventions"[All Fields] OR "interventive"[All Fields] OR "methods"[MeSH Terms] OR "methods"[All Fields] OR "intervention"[All Fields] OR "interventional"[All Fields])) OR ("feeding"[All Fields] OR "feedings"[All Fields] OR "feeds"[All Fields]) OR ("nutrition s"[All Fields] OR "nutritional status"[MeSH Terms] OR ("nutritional"[All Fields] AND "status"[All Fields]) OR "nutritional status"[All Fields] OR "nutrition"[All Fields] OR "nutritional sciences"[MeSH Terms] OR ("nutritional"[All Fields] AND "sciences"[All Fields]) OR "nutritional sciences"[All Fields] OR "nutritional"[All Fields] OR "nutritionals"[All Fields] OR "nutritions"[All Fields] OR "nutritive"[All Fields])) AND ("necrotising enterocolitis"[All Fields] OR "enterocolitis, necrotizing"[MeSH Terms] OR ("enterocolitis"[All Fields] AND "necrotizing"[All Fields]) OR "necrotizing enterocolitis"[All Fields] OR ("necrotizing"[All Fields] AND "enterocolitis"[All Fields]) OR "NEC"[All Fields])
alkaline phosphatase	("alkaline phosphatase"[MeSH Terms] OR ("alkaline"[All Fields] AND "phosphatase"[All Fields]) OR "alkaline phosphatase"[All Fields] OR "ALP"[All Fields]) AND ("necrotising enterocolitis"[All Fields] OR "enterocolitis, necrotizing"[MeSH Terms] OR ("enterocolitis"[All Fields] AND "necrotizing"[All Fields]) OR "necrotizing enterocolitis"[All Fields] OR ("necrotizing"[All Fields] AND "enterocolitis"[All Fields]) OR "NEC"[All Fields])
epidermal growth factor<br><br>heparin-binding EGF like growth factor	("EGF"[All Fields] OR ("epidermal growth factor"[MeSH Terms] OR ("epidermal"[All Fields] AND "growth"[All Fields] AND "factor"[All Fields]) OR "epidermal growth factor"[All Fields]) OR ("heparin binding egf like growth factor"[MeSH Terms] OR ("heparin binding"[All Fields] AND "egf like"[All Fields] AND "growth"[All Fields] AND "factor"[All Fields]) OR "heparin binding egf like growth factor"[All Fields] OR ("hb"[All Fields] AND "EGF"[All Fields]) OR "hb egf"[All Fields]) OR ("heparin binding egf like growth factor"[MeSH Terms] OR ("heparin binding"[All Fields] AND "egf like"[All Fields] AND "growth"[All Fields] AND "factor"[All Fields]) OR "heparin binding egf like growth factor"[All Fields] OR ("heparin"[All Fields] AND "binding"[All Fields] AND "EGF"[All Fields] AND "like"[All Fields] AND "growth"[All Fields] AND "factor"[All Fields]) OR "heparin binding egf like growth factor"[All Fields])) AND ("necrotising enterocolitis"[All Fields] OR "enterocolitis, necrotizing"[MeSH Terms] OR ("enterocolitis"[All Fields] AND "necrotizing"[All Fields]) OR "necrotizing enterocolitis"[All Fields] OR ("necrotizing"[All Fields] AND "enterocolitis"[All Fields]) OR "NEC"[All Fields])
erythropoietin	("erythropoietin"[MeSH Terms] OR "erythropoietin"[All Fields] OR "epoetin alfa"[MeSH Terms] OR ("epoetin"[All Fields] AND "alfa"[All Fields]) OR "epoetin alfa"[All Fields] OR "erythropoietins"[All Fields] OR "erythropoietin s"[All Fields] OR "EPO"[All

	Fields]) AND ("necrotising enterocolitis"[All Fields] OR "enterocolitis, necrotizing"[MeSH Terms] OR ("enterocolitis"[All Fields] AND "necrotizing"[All Fields]) OR "necrotizing enterocolitis"[All Fields] OR ("necrotizing"[All Fields] AND "enterocolitis"[All Fields]) OR "NEC"[All Fields])
exosomes	("exosomal"[All Fields] OR "exosomes"[MeSH Terms] OR "exosomes"[All Fields] OR "exosome"[All Fields] OR "exosomic"[All Fields] OR ("extracellular vesicles"[MeSH Terms] OR ("extracellular"[All Fields] AND "vesicles"[All Fields]) OR "extracellular vesicles"[All Fields]) OR ("cell derived microparticles"[MeSH Terms] OR ("cell derived"[All Fields] AND "microparticles"[All Fields]) OR "cell derived microparticles"[All Fields] OR "microvesicle"[All Fields] OR "microvesicles"[All Fields])) AND ("necrotising enterocolitis"[All Fields] OR "enterocolitis, necrotizing"[MeSH Terms] OR ("enterocolitis"[All Fields] AND "necrotizing"[All Fields]) OR "necrotizing enterocolitis"[All Fields] OR ("necrotizing"[All Fields] AND "enterocolitis"[All Fields]) OR "NEC"[All Fields])
ganglioside	("gangliosides"[MeSH Terms] OR "gangliosides"[All Fields] OR "ganglioside"[All Fields] OR "gangliosidic"[All Fields] OR "GD3"[All Fields] OR "GM"[All Fields]) AND ("necrotising enterocolitis"[All Fields] OR "enterocolitis, necrotizing"[MeSH Terms] OR ("enterocolitis"[All Fields] AND "necrotizing"[All Fields]) OR "necrotizing enterocolitis"[All Fields] OR ("necrotizing"[All Fields] AND "enterocolitis"[All Fields]) OR "NEC"[All Fields])
glutamine	("Gln"[All Fields] OR ("glutamine"[MeSH Terms] OR "glutamine"[All Fields] OR "l glutamine"[All Fields]) OR ("glutamin"[All Fields] OR "glutamine"[MeSH Terms] OR "glutamine"[All Fields] OR "glutamine s"[All Fields] OR "glutamines"[All Fields])) AND ("necrotising enterocolitis"[All Fields] OR "enterocolitis, necrotizing"[MeSH Terms] OR ("enterocolitis"[All Fields] AND "necrotizing"[All Fields]) OR "necrotizing enterocolitis"[All Fields] OR ("necrotizing"[All Fields] AND "enterocolitis"[All Fields]) OR "NEC"[All Fields])
immunoglobulins	("immunoglobulin s"[All Fields] OR "immunoglobuline"[All Fields] OR "immunoglobulines"[All Fields] OR "immunoglobulins"[MeSH Terms] OR "immunoglobulins"[All Fields] OR "immunoglobulin"[All Fields] OR ("immunoglobulin g"[MeSH Terms] OR "immunoglobulin g"[All Fields] OR "igg"[All Fields]) OR ("immunoglobulin a"[MeSH Terms] OR "immunoglobulin a"[All Fields] OR "iga"[All Fields])) AND ("necrotising enterocolitis"[All Fields] OR "enterocolitis, necrotizing"[MeSH Terms] OR ("enterocolitis"[All Fields] AND "necrotizing"[All Fields]) OR "necrotizing enterocolitis"[All Fields] OR ("necrotizing"[All Fields] AND "enterocolitis"[All Fields]) OR "NEC"[All Fields])
insulin like growth factor	("somatomedins"[MeSH Terms] OR "somatomedins"[All Fields] OR ("insulin"[All Fields] AND "like"[All Fields] AND "growth"[All Fields] AND "factor"[All Fields]) OR "insulin like growth factor"[All Fields] OR "ILGF1"[All Fields] OR "IGF1"[All Fields]) AND ("necrotising enterocolitis"[All Fields] OR "enterocolitis, necrotizing"[MeSH Terms] OR ("enterocolitis"[All Fields] AND "necrotizing"[All Fields]) OR "necrotizing enterocolitis"[All Fields] OR ("necrotizing"[All Fields] AND "enterocolitis"[All Fields]) OR "NEC"[All Fields])
milk fat globule membrane	((("milk fat globule"[Supplementary Concept] OR "milk fat globule"[All Fields]) AND ("membranal"[All Fields] OR "membrane s"[All Fields] OR "membraneous"[All Fields] OR "membranes"[MeSH Terms] OR "membranes"[All Fields] OR "membrane"[All Fields] OR "membranous"[All Fields])) OR ("milk fat globule"[Supplementary Concept] OR "milk fat globule"[All Fields])) AND

	("necrotising enterocolitis"[All Fields] OR "enterocolitis, necrotizing"[MeSH Terms] OR ("enterocolitis"[All Fields] AND "necrotizing"[All Fields]) OR "necrotizing enterocolitis"[All Fields] OR ("necrotizing"[All Fields] AND "enterocolitis"[All Fields]) OR "NEC"[All Fields])
oligosaccharides	("gos"[All Fields] OR "FOS"[All Fields] OR ("oligosaccharides"[MeSH Terms] OR "oligosaccharides"[All Fields] OR "oligosaccharide"[All Fields] OR "oligosaccharidic"[All Fields]) AND ("necrotising enterocolitis"[All Fields] OR "enterocolitis, necrotizing"[MeSH Terms] OR ("enterocolitis"[All Fields] AND "necrotizing"[All Fields]) OR "necrotizing enterocolitis"[All Fields] OR ("necrotizing"[All Fields] AND "enterocolitis"[All Fields]) OR "NEC"[All Fields])
osteopontin	("osteopontin"[MeSH Terms] OR "osteopontin"[All Fields] OR "osteopontine"[All Fields] OR "osteopontins"[All Fields] OR ("opt photonics news"[Journal] OR "opn"[All Fields])) AND ("necrotising enterocolitis"[All Fields] OR "enterocolitis, necrotizing"[MeSH Terms] OR ("enterocolitis"[All Fields] AND "necrotizing"[All Fields]) OR "necrotizing enterocolitis"[All Fields] OR ("necrotizing"[All Fields] AND "enterocolitis"[All Fields]) OR "NEC"[All Fields])
platelet-activating factor acetylhydrolase	("PAF-AH"[All Fields] OR (("platelet activating factor"[MeSH Terms] OR ("platelet"[All Fields] AND "activating"[All Fields] AND "factor"[All Fields]) OR "platelet activating factor"[All Fields]) AND ("acetylhydrolase"[All Fields] OR "acetylhydrolases"[All Fields]))) AND ("necrotising enterocolitis"[All Fields] OR "enterocolitis, necrotizing"[MeSH Terms] OR ("enterocolitis"[All Fields] AND "necrotizing"[All Fields]) OR "necrotizing enterocolitis"[All Fields] OR ("necrotizing"[All Fields] AND "enterocolitis"[All Fields]) OR "NEC"[All Fields])
polyunsaturated fatty acid	("PUFA"[All Fields] OR ("fatty acids, unsaturated"[MeSH Terms] OR ("fatty"[All Fields] AND "acids"[All Fields] AND "unsaturated"[All Fields]) OR "unsaturated fatty acids"[All Fields] OR ("polyunsaturated"[All Fields] AND "fatty"[All Fields] AND "acid"[All Fields]) OR "polyunsaturated fatty acid"[All Fields]) OR ("fatty acids, omega 3"[MeSH Terms] OR ("fatty"[All Fields] AND "acids"[All Fields] AND "omega 3"[All Fields]) OR "omega-3 fatty acids"[All Fields] OR "omega 3 fatty acids"[All Fields]) OR ("fatty acids, omega 6"[MeSH Terms] OR ("fatty"[All Fields] AND "acids"[All Fields] AND "omega 6"[All Fields]) OR "omega-6 fatty acids"[All Fields] OR "omega 6 fatty acids"[All Fields]) OR ("long"[All Fields] AND ("chain"[All Fields] OR "chain s"[All Fields] OR "chains"[All Fields]) AND ("fatty acids"[MeSH Terms] OR ("fatty"[All Fields] AND "acids"[All Fields]) OR "fatty acids"[All Fields]))) AND ("necrotising enterocolitis"[All Fields] OR "enterocolitis, necrotizing"[MeSH Terms] OR ("enterocolitis"[All Fields] AND "necrotizing"[All Fields]) OR "necrotizing enterocolitis"[All Fields] OR ("necrotizing"[All Fields] AND "enterocolitis"[All Fields]) OR "NEC"[All Fields])
transforming growth factor β	("transforming growth factor beta"[MeSH Terms] OR ("transforming"[All Fields] AND "growth"[All Fields] AND "factor"[All Fields] AND "beta"[All Fields]) OR "transforming growth factor beta"[All Fields] OR "tgfbeta1"[All Fields] OR ("tgfbeta s"[All Fields] OR "tgfbetas"[All Fields] OR "transforming growth factor beta"[MeSH Terms] OR ("transforming"[All Fields] AND "growth"[All Fields] AND "factor"[All Fields] AND "beta"[All Fields]) OR "transforming growth factor beta"[All Fields] OR "tgfbeta"[All Fields])) AND ("necrotising enterocolitis"[All Fields] OR "enterocolitis, necrotizing"[MeSH Terms] OR ("enterocolitis"[All Fields] AND "necrotizing"[All Fields]) OR "necrotizing enterocolitis"[All Fields] OR ("necrotizing"[All Fields] AND "enterocolitis"[All Fields]) OR "NEC"[All Fields])

vitamin A	("vitamin a"[MeSH Terms] OR "vitamin a"[All Fields] OR ("tretinoin"[MeSH Terms] OR "tretinoin"[All Fields] OR ("trans"[All Fields] AND "retinoic"[All Fields] AND "acid"[All Fields]) OR "all trans retinoic acid"[All Fields]) OR ("vitamin a"[MeSH Terms] OR "vitamin a"[All Fields] OR "retinol"[All Fields] OR "retinols"[All Fields]) OR ("retinoidal"[All Fields] OR "retinoids"[MeSH Terms] OR "retinoids"[All Fields] OR "retinoid"[All Fields])) AND ("necrotising enterocolitis"[All Fields] OR "enterocolitis, necrotizing"[MeSH Terms] OR ("enterocolitis"[All Fields] AND "necrotizing"[All Fields]) OR "necrotizing enterocolitis"[All Fields] OR ("necrotizing"[All Fields] AND "enterocolitis"[All Fields]) OR "NEC"[All Fields])
vitamin D	("vitamin d"[MeSH Terms] OR "vitamin d"[All Fields] OR "ergocalciferols"[MeSH Terms] OR "ergocalciferols"[All Fields] OR ("cholecalciferol"[MeSH Terms] OR "cholecalciferol"[All Fields] OR "cholecalciferols"[All Fields] OR "colecalciferol"[All Fields]) OR ("ergocalciferols"[MeSH Terms] OR "ergocalciferols"[All Fields] OR "ergocalciferol"[All Fields]) OR "D2"[All Fields] OR "D3"[All Fields]) AND ("necrotising enterocolitis"[All Fields] OR "enterocolitis, necrotizing"[MeSH Terms] OR ("enterocolitis"[All Fields] AND "necrotizing"[All Fields]) OR "necrotizing enterocolitis"[All Fields] OR ("necrotizing"[All Fields] AND "enterocolitis"[All Fields]) OR "NEC"[All Fields])

Supplementary table S2. Embase search

# ▲	Searches	Results	Type
1	necrotizing enterocolitis/	12218	Advanced
2	nutrition/	109194	Advanced
3	feeding/	52256	Advanced
4	2 or 3	158880	Advanced
5	1 and 4	533	Advanced
6	limit 5 to yr="1883 - 2020"	513	Advanced
7	erythropoietin/	36171	Advanced
8	1 and 7	105	Advanced
9	limit 8 to yr="1883 - 2020"	102	Advanced
10	alkaline phosphatase/	110896	Advanced
11	1 and 10	79	Advanced
12	limit 11 to yr="1883 - 2020"	79	Advanced
13	glutamine/	41784	Advanced
14	1 and 13	92	Advanced
15	limit 14 to yr="1883 - 2020"	92	Advanced
16	epidermal growth factor/	41340	Advanced
17	heparin binding epidermal growth factor/	2781	Advanced
18	16 or 17	43596	Advanced
19	1 and 18	161	Advanced
20	limit 19 to yr="1883 - 2020"	160	Advanced
21	exosome/	32781	Advanced
22	1 and 21	33	Advanced
23	limit 22 to yr="1883 - 2020"	30	Advanced
24	ganglioside/	9435	Advanced
25	1 and 24	8	Advanced

☐	26	limit 25 to yr="1883 - 2020"	8	Advanced
☐	27	somatomedin/	16114	Advanced
☐	28	1 and 27	17	Advanced
☐	29	limit 28 to yr="1883 - 2020"	17	Advanced
☐	30	milk fat/	2925	Advanced
☐	31	1 and 30	10	Advanced
☐	32	limit 31 to yr="1883 - 2020"	9	Advanced
☐	33	osteopontin/	17679	Advanced
☐	34	1 and 33	8	Advanced
☐	35	limit 34 to yr="1883 - 2020"	8	Advanced
☐	36	oligosaccharide/	25624	Advanced
☐	37	1 and 36	113	Advanced
☐	38	limit 37 to yr="1883 - 2020"	110	Advanced
☐	39	polyunsaturated fatty acid/	22681	Advanced
☐	40	1 and 39	62	Advanced
☐	41	limit 40 to yr="1883 - 2020"	61	Advanced
☐	42	retinoic acid/	43088	Advanced
☐	43	1 and 42	12	Advanced
☐	44	limit 43 to yr="1883 - 2020"	12	Advanced
☐	45	transforming growth factor beta/	86269	Advanced
☐	46	1 and 45	59	Advanced
☐	47	limit 46 to yr="1883 - 2020"	58	Advanced
☐	48	vitamin D/	79604	Advanced
☐	49	1 and 48	57	Advanced
☐	50	limit 49 to yr="1883 - 2020"	56	Advanced

☐	51	immunoglobulin/	120054	Advanced
☐	52	1 and 51	168	Advanced
☐	53	limit 52 to yr="1945 - 2020"	168	Advanced
☐	54	1 alkyl 2 acetylglycerophosphocholine esterase/	2314	Advanced
☐	55	1 and 54	19	Advanced
☐	56	limit 55 to yr="1883 - 2020"	19	Advanced

Supplementary table S3. Cochrane library search

#1	feeding intervention	S	MeSH	Limits	9442
#2	nutritional intervention			Limits	13452
#3	feeding			Limits	23499
#4	nutrition			Limits	55842
#5	necrotizing enterocolitis			Limits	1999
#6	NEC			Limits	998
#7	#1 OR #2 OR #3 OR #4			Limits	77042
#8	#5 OR #6			Limits	2344
#9	#7 AND #8			Limits	922
#10	#7 AND #8<br>with Cochrane Library publication date to Jan 2021, in Cochrane Reviews			Limits	185
#11	erythropoietin			Limits	4358
#12	EPO			Limits	2290
#13	#11 OR #12			Limits	4821
#14	#13 AND #8			Limits	44
#15	#13 AND #8<br>with Cochrane Library publication date to Jan 2021, in Cochrane Reviews			Limits	11
#16	alkaline phosphatase			Limits	5916
#17	ALPI			Limits	30
#18	#16 OR #17			Limits	5946
#19	#18 AND #8			Limits	30
#20	#18 AND #8<br>with Cochrane Library publication date to Jan 2021, in Cochrane Reviews			Limits	13

#21	Gln	Limits	325
#22	L-glutamine	Limits	241
#23	glutamine	Limits	2098
#24	#21 OR #22 OR #23	Limits	2159
#25	#24 AND #8	Limits	27
#26	#24 AND #8<br>with Cochrane Library publication date to Jan 2021, in Cochrane Reviews	Limits	7
#27	EGF	Limits	603
#28	epidermal growth factor	Limits	4834
#29	HB-EGF	Limits	16
#30	heparin-binding EGF like growth factor	Limits	10
#31	#27 OR #28 OR #29 OR #30	Limits	5067
#32	#31 AND #8	Limits	6
#33	#31 AND #8<br>with Cochrane Library publication date to Jan 2021, in Cochrane Reviews	Limits	2
#34	exosomes	Limits	106
#35	extracellular vesicles	Limits	118
#36	microvesicles	Limits	48
#37	#34 OR #35 OR #36	Limits	250
#38	#37 AND #8	Limits	2
#39	#37 AND #8<br>with Cochrane Library publication date to Jan 2021, in Cochrane Reviews	Limits	0
#40	ganglioside	Limits	231

#41	GD3	Limits	27
#42	GM	Limits	15436
#43	#40 OR #41 OR #42	Limits	15654
#44	#43 AND #8	Limits	59
#45	#43 AND #8<br>with Cochrane Library publication date to Jan 2021, in Cochrane Reviews	Limits	16
#46	insulin like growth factor	Limits	3538
#47	ILGF1	Limits	0
#48	IGF1	Limits	245
#49	#46 OR #47 OR #48	Limits	3667
#50	#49 AND #8	Limits	29
#51	#49 AND #8<br>with Cochrane Library publication date to Jan 2021, in Cochrane Reviews	Limits	22
#52	milk fat globule membrane	Limits	61
#53	milk fat globule	Limits	78
#54	#52 OR #53	Limits	78
#55	#54 AND #8	Limits	2
#56	#54 AND #8<br>with Cochrane Library publication date to Jan 2021, in Cochrane Reviews	Limits	2
#57	osteopontin	Limits	232
#58	OPN	Limits	273
#59	#57 OR #58	Limits	429
#60	#59 AND #8	Limits	1

#61	#59 AND #8<br>with Cochrane Library publication date to Jan 2021, in Cochrane Reviews	Limits	0
#62	GOS	Limits	894
#63	FOS	Limits	505
#64	oligosaccharides	Limits	1012
#65	HMO	Limits	427
#66	#62 OR #63 OR #64 OR #65	Limits	2424
#67	#66 AND #8	Limits	30
#68	#66 AND #8<br>with Cochrane Library publication date to Jan 2021, in Cochrane Reviews	Limits	13
#69	PUFA	Limits	2024
#70	polyunsaturated fatty acid	Limits	2790
#71	omega 3 fatty acids	Limits	4973
#72	omega 6 fatty acids	Limits	2924
#73	long chain fatty acids	Limits	1748
#74	#69 OR #70 OR #71 OR #72 OR #73	Limits	7515
#75	#74 AND #8	Limits	33
#76	#74 AND #8<br>with Cochrane Library publication date to Jan 2021, in Cochrane Reviews	Limits	14
#77	vitamin A	Limits	27965
#78	all trans retinoic acid	Limits	413
#79	retinol	Limits	2262
#80	retinoids	Limits	504

#81	#77 OR #78 OR #79 OR #80	Limits	29410
#82	#81 AND #8	Limits	109
#83	#81 AND #8<br>with Cochrane Library publication date to Jan 2021, in Cochrane Reviews	Limits	44
#84	transforming growth factor beta	Limits	869
#85	TGFbeta1	Limits	234
#86	TGFbeta	Limits	447
#87	#84 OR #85 OR #86	Limits	1205
#88	#87 AND #8	Limits	2
#89	#87 AND #8<br>with Cochrane Library publication date to Jan 2021, in Cochrane Reviews	Limits	1
#90	vitamin D	Limits	17384
#91	cholecalciferol	Limits	3066
#92	ergocalciferol	Limits	354
#93	D2	Limits	4229
#94	D3	Limits	5590
#95	#90 OR #91 OR #92 OR #93 OR #94	Limits	22866
#96	#95 AND #8	Limits	76
#97	#95 AND #8<br>with Cochrane Library publication date to Jan 2021, in Cochrane Reviews	Limits	35
#98	immunoglobulins	Limits	2939
#99	IgG	Limits	5645
#100	IgA	Limits	4221

#101	#98 OR #99 OR #100	Limits	10801
#102	#101 AND #8	Limits	58
#103	#101 AND #8<br>with Cochrane Library publication date to Jan 2021, in Cochrane Reviews	Limits	22
#104	PAF-AH	Limits	24
#105	platelet-activating factor acetylhydrolase	Limits	28
#106	#104 OR #105	Limits	33
#107	#106 AND #8	Limits	0
#108	#106 AND #8<br>with Cochrane Library publication date to Jan 2021, in Cochrane Reviews	Limits	0

Supplementary table S4. Overview of RCTs that were not incorporated in one of the included meta-analyses and had a too small sample size (<50% of infants in the meta-analysis on the same intervention) to be included as separate trial.

Author and year	Type of study and sample size	In- and exclusion criteria	Control	Intervention	Effect on NEC incidence
Cui et al.<br><br>2019	single center RCT<br>N=114 neonates	Inclusion: formula fed preterm infants admitted within 12h after birth to the First Neonatal Ward of the Shengjing Hospital of China Medical University, gestational age ≥ 30 and < 37 weeks; birthweight ≥1500 g and ≤ 2000 g, vital sign and hemodynamic parameters stable.<br><br>Exclusion: congenital diseases, expected hospitalization <2 weeks, maternal or neonatal antibiotics or probiotics administration before admission	no probiotics (N=57 neonates)	*Lactobacillus reuteri* DSM 17938, $1*10^8$ CFU (5 drops) once daily, beginning with the first feeding until discharge from the hospital, *Lactobacillus reuteri* administration was stopped if enteral feeding was stopped due to feeding intolerance and resumed after feedings resumed<br>(N=57 neonates)	NEC incidence control group 10.42%<br>NEC incidence intervention group 2.22% (NS)
Gómez-Rodríguez et al.<br><br>2019	single center RCT<br>N=90 neonates	Inclusion: preterm newborns, birthweight between 700 and 1500 g and gestational age <33 weeks, no counter indication for enteral feeding in the first 7 days postntatally, inborn in tertiary healthcare from January 2014 to May 2015.<br><br>Exclusion: birthweight >1500 gram, Apgar score <6 at 5 minutes, gastrointestinal	single strain probiotic containing $1*10^9$ CFU *Lactobacillus acidophilus boucardi* mixed in the feed each 24h during three weeks, starting from	multispecies probiotic containing $1*10^9$ CFU *Lactobacillus acidophilus,* $4.4*10^8$ CFU *Lactobacillus rhamnosus,* $1*10^9$ CFU *Lactobacillus casei*, $1.76*10^8$ CFU *Lactobacillus plantroom,* $2.76*10^7$ CFU Bifidobacterium infantis and $6.6*10^5$ CFU *Streptococcus thermophiles* mixed in the feed each 24h	1 infant developed NEC in the multispecies probiotic group, no infants developed NEC in the single strain probiotics group

		malformations, patent ductus arteriosus with hemodynamic alterations and septic shock	day 5 (N=45 neonates)	during three weeks, starting from day 5 (N=45 neonates)	
Maamouri et al.<br><br>2016	single center RCT N=105 neonates	Inclusion: preterm neonates, gestational age <32 weeks, birth weight <1500 g, Apgar scores >7 at the NICU of the Qaem Hospital affiliated to the Mashhad University of Medical Sciences, Masshad, Iran, informed consent<br><br>Exclusion: discharge before the end of the treatment period, intraventricular hemorrhage, metabolic disease or congenital anomalies	no enteral administration of L-glutamine (N=53 neonates)	L-glutamine, 0.3 g/kg/day divided in three doses, 5% solution, enterally administered from the 3rd until the 28th day of life (N=52 neonates)	OR 0.943 (95% CI 0.883-1.008) (NEC incidence)
Tarnow-Mordi<br><br>2020	multi center RCT N=1542 neonates	Inclusion: birth weight <1500 g, age <8 days<br><br>Exclusion: lethal anomalies, late-onset sepsis before consent,	no pasteurized bovine lactoferrin added to feeds (N=771 neonates)	pasteurized bovine lactoferrin, 200 mg/kg, once daily added to formula feeding or breast milk, starting immediately after randomization and continued until 34 weeks post-mentstrual age or for two weeks (whichever was longer) (N=771 neonates)	RR 1.09 (95% CI 0.63-1.9) (incidence stage II or III NEC)

Supplementary table S5. Overview included experimental animal studies on enteral feeding interventions for prevention of NEC

Author and year	Model used	Control	Intervention	Sample size	Sample size / power calculation	Outcomes studied
Akisü et al. 1998	Balb/c mice (25-30 days old), NEC induction with one time hypoxia-reoxygenation (100% CO_2 for 5 minutes followed by 100% O_2 for 10 minutes), sacrifice after 4 hours	standard mouse chow (triglyceride composition: 45% saturated fatty acids, 40 % monounsaturated fatty acids, 15% n-6 PUFA, 0% n-3 PUFA)	10% w/w fish oil supplemented mouse chow (triglyceride composition: 40% saturated fatty acids, 30% monounsaturated fatty acids, 18% EPA, 12% DHA), for 4 weeks prior to NEC induction	18 mice (N=6 per group)	not provided	histological NEC incidence/severity<br><br>intestinal inflammation
Akisü et al. 2002	Balb/c mice (25-30 days old), NEC induction with one time hypoxia-reoxygenation (100% CO_2 for 5 minutes followed by 100% O_2 for 10 minutes), sacrifice after 4 hours	physiological saline, 1 mL, by intraperitoneal injection immediately before NEC induction	L-arginine capsule in drinking water, 2 g/L, for 7 days prior to NEC induction<br><br>L-carnitine solution in water, 50 mg/kg orally, for 7 days prior to NEC induction	28 mice (N=7 per group)	not provided	histological NEC incidence/severity<br><br>vascular function / hypoxia-ischemia / free radical formation
Akisü et al. 2003	Balb/c mice (25-30 days old), NEC induction with one time hypoxia-reoxygenation (100% CO_2 for 5 minutes followed by 100% O_2 for 10 minutes), sacrifice after 4 hours	physiologic saline, 1 mL, by orogastric intubation immediately before NEC induction	L-glutamine in drinking water, 0.5 g/dL, for 3 days before NEC induction<br><br>L-glutamine in drinking water, 3 g/dL, for 10 days before NEC induction	32 mice (N=8 per group)	not provided	histological NEC incidence/severity<br><br>intestinal inflammation

Autran et al. 2016	Sprague-Dawley rats (newborn), NEC induction with formula feeding (200 μL twice daily) and hypoxia (5% O_2, 95% N2 for 10 minutes) thrice daily for 4 days	Formula without HMO, GOS or sialylated GOS	HMO in formula, 10 mg/ml, simultaneous with NEC induction<br><br>GOS in formula, 8 mg/ml, simultaneous with NEC induction<br><br>sialylated GOS in formula, 500 μM, simultaneous with NEC induction<br><br>2'-FL in formula, 2 mg/ml, simultaneous with NEC induction	95 rats (N=11-22 per group)	not provided	histological NEC incidence/severity
Bergmann et al. 2013	C57BL/6 mice (newborn), NEC induction with inoculation with a standardized adult commensal bacteria mixture (10^7 CFU) within 12 hours after birth, formula feeding (30 μL every 3 hours) and asphyxia (100% N_2 for 1 minute) and cold exposure (4°C for 10 minutes) twice daily for 3 days	inoculation with vehicle	*Bifidobacterium infantis* strain BB-02 (human source), $3x10^6$ CFU in 20 μL in dextrose (immunofluorescence studies) or maltodextran (permability studies, Western blot, histology), before NEC induction	not provided	not provided	histological NEC incidence/severity<br>,<br><br>intestinal barrier function
Butel et al. 1998	Germ-free quails (*coturnix, coturnix* subsp. j*aponica)* (2 week old), NEC induction with colonization with fecal flora isolated from an infant with NEC and feeding with a 6-8% w/w lactose diet for 21 days	inoculation with a 10-fold dilution of fecal flora in 100 μL	*Bifidobacterium infantis-longum* strain CUETM 89-215 isolated from stool of a healthy premature infant, 10^8 viable cells/ml, 100 μL, simultaneous with NEC induction	59 quails (N=8-12 per group)	not provided	histological NEC incidence/severity<br><br>microbiome alterations
Cai et al. 2016	Sprague-Dawley rats (15 days old), NEC induction with asphyxia (100% N_2 for 2 minutes) and cold exposure (4°C	oral saline	astragaloside IV 25 mg/kg/d orally, simultaneous with NEC induction	40 rats (N=10 per group)	not provided	histological NEC incidence/severity<br><br>intestinal

	for 10 minutes) twice daily for 4 days		astragaloside IV 50 mg/kg/d orally, simultaneous with NEC induction<br><br>astragaloside IV 75 mg/kg/d orally, simultaneous with NEC induction			inflammation<br><br>systemic inflammation<br><br>vascular function / hypoxia-ischemia / free radical formation
Cakir et al. 2018	Wistar rats (newborn), NEC induction with one time intraperitoneal LPS (1 mg/kg) on day 1, formula feeding (200 μL every 3h and increased by 100 μL daily if tolerated) and asphyxia (100% CO_2 for 10 minutes), hyperoxia (97% O_2 for 5 minutes) and cold exposure (4°C for 10 minutes) twice daily for 3 days	saline, 2 ml/kg, once daily, oral	ginger extract in water, 1000 mg/kg, once daily, simultaneous with NEC induction	30 rats (N=10 per group)	not provided	histological NEC incidence/severity<br><br>NEC signs and symptoms<br><br>intestinal inflammation<br><br>vascular function / hypoxia-ischemia / free radical formation<br><br>intestinal epithelial cell death
Caplan et al. 1997	Sprague-Dawley rats (newborn), NEC induction with formula feeding (100 μL every 3h and increased to 400 μL on day 4 if tolerated) and asphyxia (100% N_2 for 50 seconds) followed by	25 μL dilution buffer in each feeding via orogastric tube	rPAF-AH, 80 U in 25 μL dilution buffer in each feeding via orogastric tube, the PAF-AH preparation had 0.8 mg/ml protein and approximately 4000 U/mg PAF-AH activity	52 rats (N=26 per group)	not provided	histological NEC incidence/severity<br><br>NEC survival

	cold exposure (4°C for 10 minutes) twice daily for 4 days					
Caplan et al. 1999	Sprague-Dawley rats (newborn on day 21 of gestation), NEC induction with formula feeding (100 μL every 3h and increased to 400 μL on day 4 if tolerated) and asphyxia (100% N_2 for 50 seconds) followed by cold exposure (4°C for 10 minutes) twice daily for 4 days	no administration of *Bifidobacterium infantis*.<br><br>a second control group was used with administration of *Escherichia coli*, 10^9 organisms, once daily, 30 minutes before asphyxia via orogastric tube	*Bifidobacterium infantis* (human source), 10^9 organisms, once daily, 30 minutes before asphyxia via orogastric tube, simultaneous with NEC induction	51 rats (N=24-27 per group)	not provided	histological NEC incidence/severity<br><br>NEC survival<br><br>intestinal inflammation<br><br>intestinal barrier function
Caplan et al. 2001	Sprague-Dawley rats (newborn on day 21 of gestation), NEC induction with formula feeding (100 μL every 3h and increased to 300 μL on day 3 if tolerated) and asphyxia (100% N_2 for 50 seconds) followed by cold exposure (4°C for 10 minutes) twice daily for 3 days	formula without PUFA and nucleotide supplementation (CMP 3.55 mg/100 g formula powder/200 mL water, UMP 6.2 mg/100 g formula powder/200 mL water, IMP 0.73 mg/100 g	PUFA in formula, 34 mg/100 mL AA, 23 mg/100 mL DHA, simultaneous with NEC induction<br><br>PUFA and nucleotides in formula, 34 mg/100 mL AA, 23 mg/100 mL DHA, CMP 15.62 mg/100 g formula powder/200 mL water, UMP 9.2 mg/100 g formula powder/200 mL water, IMP 3.06 mg/100 g formula powder/200 mL water, GMP 2.08 mg/100 g formula powder/200 mL water, AMP 03.57 mg/100 g formula powder/200 mL	95 rats (N=23-24 per group)	not provided	histological NEC incidence/severity<br><br>NEC survival<br><br>intestinal inflammation<br><br>intestinal barrier function<br><br>vascular function / hypoxia-ischemia / free

		formula poweder/200 mL water, GMP 0.2 mg/100 g formula poweder/200 mL water, AMP 0.18 mg/100 g formula poweder/200 mL water)	water, simultaneous with NEC induction			radical formation<br><br>intestinal epithelial cell death
Catala et al.<br><br>1999	Germ-free quails (*coturnix, coturnix* subsp. *japonica)* (2 week old), NEC induction with colonization with fecal flora isolated from an infant with NEC and feeding with a 6% w/w lactose diet for 21 days	control diet with 6% lactose w/w	diet of 3% FOS and 3% lactose w/w, simultaneous with NEC induction	80 quails (N=8-12 per group)	not provided	NEC signs and symptoms
Chen et al. 2012	Sprague-Dawley rats (newborn on day 21 of gestation), NEC induction with formula feeding and asphyxia (100% N_2 for 90 seconds) and cold exposure (4°C for 10 minutes) thrice daily for 3 days	formula without recombinant human HB-EGF	recombinant human HB-EGF in formula, 800 μg/kg/dose, simultaneous with NEC induction	25 rats (N=5-10 per group)	not provided	intestinal barrier function<br><br>intestinal epithelial proliferation<br><br>enteric nervous system
Chen et al. 2019	C57BL/6 mice (5-9 days old), NEC induction with enteral administration of LPS (4 mg/kg/day) once daily, formula feeding (50 μL/g thrice daily)	control formula	arginine in formula, 240 mg/kg/day, simultaneous with NEC induction	36 mice (N=8-11 per group)	not provided	histological NEC incidence/severity<br><br>intestinal inflammation

	and hypoxia (5% O_2 for 10 minutes) thrice daily for 4 days					vascular function / hypoxia-ischemia / free radical formation
Cigsar et al. 2018	Wistar albino rats (newborn), NEC induction with formula feeding (200 μL twice daily) and asphyxia (100% CO_2 for 10 minutes), hyperoxia (97% O_2 for 10 minutes) and cold exposure (4°C for 10 minutes) twice daily for 3 days	no oral sesamol	97% sesamol orally, 100 mg/kg/dose, twice daily, simultaneous with NEC induction	34 rats (N=7-9 per group)	not provided	histological NEC incidence/severity<br><br>NEC signs and symptoms<br><br>vascular function / hypoxia-ischemia / free radical formation<br><br>intestinal epithelial cell death
Cilieborg et al. 2016	Preterm pigs (90% of gestational age, delivered by caesarian section), NEC induction with parenteral nutrition for the first 2 postnatal days (4-6 mL/kg/h) with minimal enteral feeding (2-3 mL/kg per 3 hours) and full enteral nutrition after 2 days with an oral bolus of 15 ml/kg formula every 3 hours for 3 days	control formula	2'-FL in formula, 5 g/L, during minimal enteral feeding and full enteral feeding, simultaneous with NEC induction	33 pigs (N=16-17 per group)	Yes, power calculation based on estimated mean and dispersion for NEC incidence (69%, SEM 21%), estimated effect size of 30% and β 80%; required	histological NEC incidence/severity<br><br>NEC signs and symptoms<br><br>microbiome alterations<br><br>digestion and absorption

					sample size 17	
Clark et al. 2005	Sprague-Dawley rats (newborn), NEC induction with formula feeding (100 μL every 3-4 hours) and asphyxia (100% N_2 for 1 minute) followed by cold exposure (4°C for 10 minutes) twice daily for 4 days	control cow-milk based formula (free of growth factors)	rat EGF in cow-milk based formula, 500 ng/mL, simultaneous with NEC induction	unclear	not provided	intestinal epithelial cell death / altered proliferation
Clark et al. 2006	Sprague-Dawley rats (newborn on day 21 of gestation), NEC induction with formula feeding (150 μL every 5 hours) and asphyxia (100% N_2 for 1 minute) followed by cold exposure (4°C for 10 minutes) twice daily for 4 days	control cow-milk based formula (free of growth factors)	rat EGF in cow-milk based formula, 500 ng/mL, simultaneous with NEC induction	189 rats (N=40-76 per group)	not provided	NEC survival,<br><br>intestinal barrier function
Coursodon-Boyiddle et al. 2012	Sprague-Dawley rats (newborn on day 21 of gestation), NEC induction with formula feeding (850 μL every day in 6 feeds) and asphyxia (100% N_2 for 1 minute) and cold exposure (4°C for 10 minutes) twice daily for 4 days	rat milk formula (0.0% conjugated linoleic acid, 20.9% n-6 PUFA, 3.5% n-3 PUFA)	1.5% pomegranate seed oil in rat milk formula (2.7% conjugated linoleic acid, 20.6% n-6 PUFA, 3.3% n-3 PUFA)	60 rats (N=20 per group)	not provided	histological NEC incidence/severity<br><br>NEC survival,<br><br>intestinal inflammation<br><br>intestinal epithelial proliferation
Cuna et al. 2020	C57BL6 mice (8-11 days old), NEC induction with one time intraperitoneal LPS (5 mg/kg) 12 hours before sacrifice, formula	vehicle, 0.1 mL, once daily for 2 days before NEC induction	*Lactobacillus rhamnosus* GG, 0.1 mL and 10^7 CFU/mL, once daily for 2 days before NEC induction	unclear	not provided	intestinal inflammation<br><br>intestinal barrier

	feeding (200 µL/5 g body weight twice daily) and hypoxia (5% O_2 for 2 minutes twice daily) for 3 days					function<br><br>intestinal epithelial cell death
Dilsiz et al. 2003	Sprague-Dawley rats (newborn), NEC induction with formula feeding three times a day for 4 days	formula without supplemented glutamine	glutamine (0.31 mg/kg/day) in formula feeding, simultaneous with NEC induction	40 rats (N=10 per group)	not provided	NEC signs and symptoms<br><br>vascular function / hypoxia-ischemia / free radical formation
D'Souza et al. 2010	Sprague-Dawley rats (newborn), NEC induction with formula feeding (200 µL every three hours) for 4 days	formula without supplementation	*Saccharomyces Boulardii* 5 mg/mL in formula, simultaneous with NEC induction<br><br>GOS/FOS in formula, simultaneous with NEC induction<br><br>*Saccharomyces Boulardii* 5 mg/mL and GOS/FOS in formula, simultaneous with NEC induction	unclear	not provided	NEC signs and symptoms<br><br>NEC survival,<br><br>vascular function / hypoxia-ischemia / free radical formation
D'Souza et al. 2012	Sprague-Dawley rats (newborn), NEC induction with formula feeding (200 µL every three hours), hyperoxia (50% O2) with brief periods of hypoxia (12% O_2 for 1-2 minutes) every 4 (day 1), 5 (day 2) or 6 (day 3) hours.	formula without supplementation	*Saccharomyces Boulardii* in formula, simultaneous with NEC induction<br><br>GOS/FOS in formula, simultaneous with NEC induction<br><br>*Saccharomyces Boulardii* and GOS/FOS in formula, simultaneous with NEC induction	unclear	not provided	NEC signs and symptoms<br><br>intestinal inflammation<br><br>vascular function / hypoxia-ischemia / free radical formation

Dvorak et al. 2002	Sprague-Dawley rats (newborn), NEC induction with formula feeding and asphyxia (100% N_2 for 1 minute) followed by cold exposure (4°C for 10 minutes) twice daily for 4 days	control cow-milk based formula (free of growth factors)	rat EGF in cow-milk based formula, 500 ng/mL, simultaneous with NEC induction	60 rats	not provided	histological NEC incidence/severity<br><br>NEC signs and symptoms
Dvorak et al. 2008	Sprague-Dawley rats (newborn, 1 day before scheduled birth), NEC induction with formula feeding and asphyxia (100% N_2 for 1 minute) and cold exposure (4°C for 10 minutes) twice daily for 4 days	control cow-milk based formula (free of growth factors)	human HB-EGF in cow-milk based formula, 5, 50, 500 or 1000 ng/mL, simultaneous with NEC induction<br><br>rat EGF in cow-milk based formula, 500 ng/mL, simultaneous with NEC induction<br><br>human HB-EGF, 500 ng/mL, and rat EGF, 500 ng/m, in cow-milk based formula	103 rats (N=15-24 per group)	not provided	histological NEC incidence/severity<br><br>intestinal barrier<br><br>intestinal epithelial cell death
Egan et al. 2016	C57BL/6 mice (7-8 days old), NEC induction with administration of enteric bacteria isolated from an infant with NEC (12.5 µL stool slurry in 1 mL formula), formula feeding and hypoxia (5% O_2) twice daily for 4 days	no ATRA	ATRA, dissolved in1:1 DMSO and corn oil, 6 mg/mL and 50 µg/mouse, once daily by gavage, simultaneous with NEC induction	unclear	not provided	histological NEC incidence/severity<br><br>intestinal inflammation
Ergün et al. 2007	Wistar rats (newborn), NEC induction with formula feeding twice daily and hypoxia (5% O_2, 95% N_2), thrice daily for 3 days	formula without resveratrol	resveratrol in formula, 15 mg/kg in every feed, simultaneous with NEC induction	27 rats (N=7-10 per group)	not provided	histological NEC incidence/severity<br><br>NEC signs and symptoms<br><br>vascular function / hypoxia-

						ischemia / free radical formation
Fan et al. 2019	Sprague-Dawley rats (3-5 days old), NEC induction with Cronobacter sakazakii 1x 10^9 CFU once per day, formula feeding (200 μL twice daily) and hypoxia (5% O_2 and 95% N2 thrice daily) for 2 days	formula without *Bacteroides fragilis* strain ZY-312	*Bacteroides fragilis* strain ZY-312, 1x 10^9 CFU in 200 μL formula, once daily starting 2 days before NEC induction	40 rats (N=10 per group)	not provided	histological NEC incidence/severity<br><br>NEC signs and symptoms<br><br>NEC survival<br><br>intestinal inflammation<br><br>systemic inflammation<br><br>intestinal barrier function<br><br>vascular function / hypoxia-ischemia / free radical formation<br><br>intestinal epithelial cell death
Feng et al. 2005	Sprague-Dawley rats (newborn), NEC induction with intragastric LPS (2 mg/kg) 8 hours after birth, formula feeding every 4 hours and hypoxia (100% N_2 for 1 minute) followed by cold	no HB-EGF	HB-EGF, 600 μg/kg, every 4 hours via orogastric feeding tube, simultaneous with NEC induction	unclear	not provided	histological NEC incidence/severity<br><br>NEC survival

	exposure (10 minutes at 4°C) twice daily for 4 days					intestinal barrier function
Feng et al. doi:10.1016/j.jpedsurg.2005.10.018 2006	Sprague-Dawley rats (newborn), NEC induction with intragastric LPS (2 mg/kg) 8 hours after birth, formula feeding (100 µL every 4 hours and increased to 400 µL per feed on day 4 if tolerated) and asphyxia (100% N_2 for 1 minute) followed by cold exposure (4 °C for 10 minutes) twice daily for 4 days	no HB-EGF	HB-EGF, 600 µg/kg, every 4 hours via orogastric feeding tube, simultaneous with NEC induction	62 rats (N=10-22 per group)	not provided	histological NEC incidence/severity<br><br>NEC survival,<br><br>intestinal barrier function
Feng et al. doi:10.1016/j.jpedsurg.2005.12.020 2006	Sprague-Dawley rats (newborn), NEC induction with intragastric administration of LPS (2 mg/kg) 8 hours after birth, formula feeding (100 µL every 4 hours and increased to 400 µL per feed on day 4 if tolerated) and asphyxia (100% N_2 for 1 minute) followed by cold exposure (4 °C for 10 minutes) twice daily for 4 days	formula without HB-EGF	HB-EGF in formula, 600 µg/kg, every 4 hours via orogastric feeding tube, simultaneous with NEC induction	51 rats (N=10-21 per group)	not provided	histological NEC incidence/severity<br><br>intestinal epithelial cell death
Feng et al. 2007	Sprague-Dawley rats (newborn), NEC induction with intragastric administration of LPS (2 mg/kg) 8 hours after birth, formula feeding (100 µL every 4 hours and increased to 400 µL per feed on day 4 if tolerated) and asphyxia (100% N_2 for 1 minute)	formula without HB-EGF	HB-EGF in formula, 600 µg/kg, every 4 hours via orogastric feeding tube, simultaneous with NEC induction	110 rats (dose response experiment)<br><br>9 rats (SEM) (N=3 per group)	not provided	histological NEC<br><br>NEC survival<br><br>intestinal barrier function

	followed by cold exposure (4 °C for 10 minutes) twice daily for 4 days			30 rats (migration and proliferation) (N=10 per group)		intestinal epithelial proliferation
Good et al. 2012	C57BL/6 mice (10 days old), NEC induction with formula feeding (50 µL/g body weight, 5 times a day) and hypoxia (5% O2 95% N_2 for 10 minutes) twice daily for 4 days	no amniotic fluid	amniotic fluid, 50 µL/g, daily enteral, simultaneous with NEC induction	endotoxemia at least N=3 per group, for histology at least N=10 per group	not provided	histological NEC incidence/severity<br><br>vascular function / hypoxia-ischemia / free radical formation<br><br>intestinal epithelial proliferation
Good et al. 2014	C57BL/6 mice (7-10 days old), NEC induction with supplementation of enteric bacteria isolated from an infant with severe NEC (12.5 µL stool slurry in 1 mL formula) in formula feeding (5 times a day) and hypoxia (5% O_2, 95% N_2 for 10 minutes) twice daily for 4 days	no Lr-DNA, probiotics or CpG-DNA	Lr-DNA (microbial DNA purified from Lactobacillus rhamnosus HN001), 1 mg/kg/day by oral gavage, once daily for 4 days before NEC induction<br><br>live *Lactobacillus rhamnosus* HN001, $3*10^{11}$ CFU/kg/day by oral gavage, dose equivalent to 1 mg Lr-DNA/kg/day, once daily for 4 days before NEC induction<br><br>UV-irradiated *Lactobacillus rhamnosus* HN001, $3*10^{11}$	at least N=10 per group	not provided	histological NEC incidence/severity<br><br>NEC signs and symptoms<br><br>intestinal inflammation<br><br>vascular function / hypoxia-ischemia / free radical formation

			CFU/kg/day by oral gavage, dose equivalent to 1 mg Lr-DNA/kg/day, once daily for 4 days before NEC induction<br><br>CpG-DNA,1 mg/kg/day by oral gavage, once daily for 4 days before NEC induction			
Good et al. 2014	Yorkshire piglets (newborn at day 105-108 of gestation), NEC with supplementation of enteric bacteria isolated from an infant with surgical NEC (12.5 μL stool slurry in 1 mL formula) in formula feeding (15 mL/kg every 3 hours) for 4 days	no Lr-DNA or probiotics	Lr-DNA (microbial DNA purified from *Lactobacillus rhamnosus* HN001), 1 mg/kg/day by oral gavage, once daily, simultaneous with NEC induction<br><br>live *Lactobacillus rhamnosus* HN001, $3*10^{11}$ CFU/kg/day by oral gavage, dose equivalent to 1 mg Lr-DNA/kg/day, once daily, simultaneous with NEC induction<br><br>UV-irradiated *Lactobacillus rhamnosus* HN001, $3*10^{11}$ CFU/kg/day by oral gavage, dose equivalent to 1 mg Lr-DNA/kg/day, once daily, simultaneous with NEC induction	at least N=3 per group	not provided	histological NEC incidence/severity<br><br>NEC signs and symptoms<br><br>vascular function / hypoxia-ischemia / free radical formation
Good et al. 2016	C57BL/6 mice (7-10 days old), NEC induction with supplementation of enteric bacteria isolated from an infant with severe NEC (12.5 μL stool slurry in 1 mL formula) in	formula without HMO 2′-FL	HMO 2′-FL, 5 mg/mL of formula, 0.25 mg/g, once daily, simultaneous with NEC induction	unclear	not provided	histological NEC incidence/severity<br><br>NEC signs and symptom

	formula feeding (5 times a day) and hypoxia (5% O_2, 95% N_2 for 10 minutes) twice daily for 4 days					intestinal inflammation,<br><br>vascular function / hypoxia-ischemia / free radical formation<br><br>microbiome alterations
Gunasekaran et al. 2019	Crl:CD1(ICR) mice (14-16 days old), NEC induction with intraperitoneal injection of dithizone (33 mg/kg) diluted in ethanol/ammonium hydroxide, followed by gavage administration of $1x10^8$ CFU Klebsiella pneumonia/kg (ATCC 10031), end of experiment 16 hours after intraperitoneal injection and 10 hours after bacterial administration	no sodium hyaluronate	sodium hyaluronate (35 kDA), 15 mg/kg, once daily for 3 days prior to induction of NEC and 1h prior to bacterial administration<br><br>sodium hyaluronate (35 kDA), 30 mg/kg, once daily for 3 days prior to induction of NEC and 1h prior to bacterial administration	unclear	not provided	histological NEC incidence/severity<br><br>NEC survival<br><br>systemic inflammation<br><br>intestinal barrier function
Halpern et al. 2003	Sprague-Dawley rats (newborn), NEC induction with formula feeding and asphyxia (100% N_2 for 1 minute) followed by cold exposure (4°C for 10 minutes) twice daily for 4 days	rat milk substitute without growth factors	EGF in growth-factor-free rat milk substitute, 500 ng/mL, simultaneous with NEC induction	50 rats (N=15-18 per group	not provided	histological NEC incidence/severity<br><br>intestinal inflammation
He-Yang et al. 2020	Sprague-Dawley rats (newborn), NEC induction with formula feeding and hypoxia (5% O_2, 95% N_2 for 10 minutes) and cold	formula without sialylated HMO	sialylated HMO (39.5% 6'-SL, 28.5% 3'-SL and 6.6% DSLNT) in formula, 1500 mg/L, simultaneous with NEC induction	38 rats (N=8-15 per group)	not provided	histological NEC incidence/severity<br><br>intestinal inflammation

	exposure (4 °C for 10 minutes) thrice daily for 3 days					
Hoang et al. 2018	C57BL/6 mice (newborn), NEC induction with formula feeding (100-200 μL, 4 times a day) and hypoxia (5% O_2, 95% N_2 for 10 minutes) followed by cold exposure (4 °C for 5 minutes) thrice daily for 4 days	formula without bacterial strain	*Lactobacillus reuteri* 17938 in formula,10^6 CFU/g/day/mouse, simultaneous with NEC induction	59 mice (N=15-23 per group)	not provided	histological NEC incidence/severity<br><br>intestinal inflammation
Isani et al. 2018	Sprague-Dawley rats (newborn), NEC induction with formula feeding (200 uL, every 8 hours) and hypoxia (5% O_2, 95% N_2 for 10 minutes) after each feeding for 4 days	formula without supplementation or formula supplemented with empty soybean extract	human recombinant EGF from transgenic soybean in formula, 75 μg/kg/day, simultaneous with NEC induction	unclear	not provided	histological NEC incidence/severity<br><br>NEC survival, intestinal<br><br>intestinal barrier function<br><br>vascular function / hypoxia-ischemia / free radical formation
Jain et al. 2014	Sprague-Dawley rats (newborn at day 21.5 of gestation), NEC induction with formula feeding (200 μL starting 30 minutes after birth and every 4 hours and increased by 50 μL every 12 hours to a maximum of 300 μL) and hypoxia (5% O_2, 95% N_2 for 1 minute) followed by cold exposure (4 °C for 10 minutes) twice daily for 4 days	formula without added substances<br><br>formula with added BSA, 18 ng in total	30% v/v amniotic fluid in formula, simultaneous with NEC induction<br><br>HGF in formula, 18 ng in total, simultaneous with NEC induction	amniotic fluid supplementation: 80 rats<br><br>HGF supplementation: 58 rats	not provided	histological NEC incidence/severity<br><br>intestinal inflammation,<br><br>vascular function / hypoxia-ischemia / free radical formation

Jantscher-Krenn et al. 2012	Sprague-Dawley rats (newborn), NEC induction with formula feeding (200 μL twice daily) and hypoxia (5% O_2, 95% N_2 for 10 minutes) thrice daily for 4 days	formula without HMO	HMO in formula, 10 mg/mL, for the whole study period, simultaneous with NEC induction<br><br>HMO in formula, 10 mg/mL, after the first 24 hours of NEC induction<br><br>HMO in formula, 10 mg/mL, for the first 24 hours after NEC induction only, simultaneous with NEC induction<br><br>formula supplemented with GOS (8 mg/mL), simultaneous with NEC induction<br><br>neutral HMO-containing (zero sialic acids) formula (10 mg/mL) for the whole study period<br><br>-1 HMO (one sialic acid) in formula, 10 mg/mL, for the whole study period, simultaneous with NEC induction<br><br>-2 HMO (two sialic acid) in formula, 10 mg/mL, for the whole study period, simultaneous with NEC induction<br><br>-3 HMO (three sialic acid) in formula, 10 mg/mL, for the whole	N=8-26 rats per group	not provided	histological NEC incidence/severity<br><br>NEC signs and symptoms<br><br>NEC survival

			study period, simultaneous with NEC induction<br><br>-4 HMO (four sialic acid) in formula, 10 mg/mL, for the whole study period, simultaneous with NEC induction<br><br>DSLNT in formula, 300 μM, simultaneous with NEC induction			
Jing et al 2018	Sprague-Dawley rats (newborn on day 21 of gestation), NEC induction with oral administration of LPS (4 mg/kg/day) on postnatal day 0 and 1, formula feeding (100 μL twice daily) and asphyxia (100% N_2 for 90 seconds every 4 hours) for 4 days	formula without berberine	berberine in formula, 0.6 g/kg/day, simultaneous with NEC induction	60 rats (N=10 per group)	not provided	histological NEC incidence/severity<br><br>intestinal inflammation<br><br>systemic inflammation<br><br>intestinal barrier function
Khailova et al. doi: 10.1152/ajpgi. 00141.2009 2009	Sprague-Dawley rats (newborn, 1 day before scheduled birth), NEC induction with formula feeding (total volume 850 μL/day, 6 times a day) and asphyxia (100% N_2 for 1 minute) followed by cold exposure (4 °C for 10 minutes) twice daily for 4 days	formula without bacterial strain	*Bifidobacterium bifidum* OLB6378 in formula, 5*10[6] CFU per day, simultaneous with NEC induction	76 rats (N=16-30 per group)	not provided	histological NEC incidence/severity<br><br>intestinal inflammation,<br><br>intestinal barrier function
Khailova et al.	Sprague-Dawley rats (newborn, 1 day before scheduled birth), NEC induction with formula	formula without rat EGF	rat EGF in formula, 500 ng/mL, simultaneous with NEC induction	82 rats (N=22-36 per group)	not provided	histological NEC incidence/severity

doi: 10.1203/PDR.0b013e3181aa3198 2009	feeding (total volume 850 µL/day) and asphyxia (100% N_2 for 1 minute) followed by cold exposure (4 °C for 10 minutes) twice daily for 4 days					
Khailova et al. 2010	Sprague-Dawley rats (newborn, 1 day before scheduled birth), NEC induction with formula feeding (total volume 850 µL/day, 6 times a day) and asphyxia (100% N_2 for 1 minute) followed by cold exposure (4 °C for 10 minutes) twice daily for 4 days	Formula without *Bifidobacterium bifidum* stain OLB5378	*Bifidobacterium bifidum* strain OLB6378, $5*10^6$ CFU/mL per day in two feedings, simultaneous with NEC induction	76 rats (N=16-30 per group)<br><br>for celecoxib experiments N=8 per group	not provided	histological NEC incidence/severity<br><br>intestinal epithelial cell death
Li et al. 2019	C57BL/6 mice (5-9 days old), NEC induction with enteral administration of LPS (4 mg/kg/day) on postnatal day 6 and 7, formula feeding (50 µL/g, thrice daily) and hypoxia (5% O_2 for 10 minutes) thrice daily for 4 days	formula without bovine milk exosomes	bovine milk exosomes in formula, 1 µL/µL, simultaneous with NEC induction	27 mice (N=9 per group)	not provided	histological NEC incidence/severity<br><br>intestinal inflammation,<br><br>intestinal barrier function
Li et al. 2020	C57BL/6 mice (5-9 days old), NEC induction with enteral administration of LPS (4 mg/kg/day) on postnatal day 6 and 7, formula feeding (50 µL/g, thrice daily) and hypoxia (5% O_2 for 10 minutes) thrice daily for 4 days	formula without HMO	HMO in formula, 20 mg/ml, 2% w/v, 2-3 mg HMO/g body weight/day, simultaneous with NEC induction	N=6-15 per group	not provided	histological NEC incidence/severity<br><br>intestinal barrier function<br><br>intestinal epithelial cell death / proliferation

Liu et al. 2012	Sprague-Dawley rats (newborn), NEC induction with formula feeding (100-200 μL four times daily) and hypoxia (5% O_2, 95% N_2 for 10 minutes) thrice daily for 3 days	formula without bacterial strain	*Lactobacillus reuteri* DSM 17938 in formula, 10^6 CFU/g body weight/day, simultaneous with NEC induction<br><br>*Lactobacillus reuteri* ATCC PTA 4659 in formula, 10^6 CFU/g body weight/day, simultaneous with NEC induction	190 rats (N=17-46 per group)	not provided	histological NEC incidence/severity ,<br><br>NEC survival<br><br>intestinal inflammation
Liu et al. 2013	Sprague-Dawley rats (newborn), NEC induction with formula feeding (100-200 μL 5 times daily) and hypoxia (5% O_2, 95% N_2 for 10 minutes) thrice daily for 3 days	formula without bacterial strain	*Lactobacillus reuteri* DSM 17938 in formula, 10^6 CFU/g body weight/day, simultaneous with NEC induction	59 rats (N=15-22 per group)	not provided	NEC survival,<br><br>intestinal inflammation
Liu et al. 2014	C57BL/6 mice (8-10 days old), NEC induction with formula feeding (200 μL four times daily) and hypoxia (5% O_2, 95% N_2 for 10 minutes) followed by cold exposure (4 °C for 5 minutes) twice daily for 4 days	formula without bacterial strain	*Lactobacillus reuteri* DSM 17938 in formula, 10^6 CFU/g body weight/day, simultaneous with NEC induction	95 mice (N=16-36 per group)	not provided	histological NEC incidence/severity<br><br>NEC survival<br><br>intestinal inflammation
Liu et al. 2019	C57BL/6 mice (5-9 days old), NEC induction with administration of LPS, formula feeding and hypoxia for 4 days	no recombinant lactoferrin	recombinant lactoferrin, 0.3 g/kg/day, 6g/L, once daily by gavage from postnatal day 6 to 8	24 mice (N=7-9 per group)	not provided	histological NEC incidence/severity<br><br>intestinal inflammation<br><br>intestinal epithelial proliferation

Lock et al. 2020	Sprague-Dawley rats (newborn, 1 day before scheduled birth), NEC induction with 10^7 CFU of both Serratia marcescens, Klebsiella pneumonia and Streptococcus viridans in formula once daily, formula feeding (100 μL every 3 hours and increased to 250 μL over 5 days if tolerated) and hypoxia (5% O_2, 95% N_2 for 10 minutes) every 8 hours for 5 days	formula without supplementation	egg white lysozyme in formula, 0.37 mg in 100 μL, 0.82 mg in 250 μL, simultaneous with NEC induction<br><br>DHA in formula, 0.27 μL in 100 μL, 0.63 μL in 250 μL, simultaneous with NEC induction	60 rats (N=15-30 per group)	not provided	NEC survival,<br><br>intestinal barrier function
Lu et al. 2007	Sprague-Dawley rats (newborn on day 21 of gestation), NEC induction with formula feeding (100 μL every 3 hours and increased to 300 μL every 3 hours over 3 days) and asphyxia (100% N_2 for 50 seconds twice daily) for 3 days	formula without long-chain PUFA supplementation	AA and DHA in formula, AA 0.7% of total fatty acids, DHA 0.5% of total fatty acids, simultaneous with NEC induction<br><br>egg phospholipids in formula, 0.7% AA of total fatty acids, 0.5% DHA of total fatty acids, simultaneous with NEC induction<br><br>DHA in formula, 0.5% DHA of total fatty acids, simultaneous with NEC induction	352 rats (N=85-90 per group)	Yes, power calculation based on estimated NEC incidence (60%), estimated effect size of 50%, α 0.05 and β 0.8; required sample size 90	histological NEC incidence/severity<br><br>intestinal inflammation
Lu et al. article in Chinese 2018	Sprague-Dawley rats (newborn on day 21 of gestation), NEC induction with formula feeding (100 μL every 4 hours and increased to 300 μL every 3	formula without *Bifidobacterium* mixture	*Bifidobacterium* mixture (*Bifidobacterium adolescentis*, *Bifidobacterium breve* and *Bifidobacterium bidifum*) in formula, $1.5*10^{10}$ CFU/ml, via gastric tube,	40 rats (N=10 per group)	not provided	histological NEC incidence/severity<br><br>intestinal barrier function

	hours over 3 days), hypoxia (<1% O_2 for 1 minute) followed by cold exposure (4 °C for 10 minutes) twice daily for 4 days		once a day after cold exposure, simultaneous with NEC induction			
Lyu et al. 2020	C57BL/6 mice (5-6 weeks old), NEC induction with formula feeding and hypoxia (5% O_2, 95% N_2 for 1 minute) followed by cold exposure (4 °C for 10 minutes) within 1 hour after feeding twice daily for 4 days	saline by gavage	vitamin D by gavage, 0.5 g/kg/day, simultaneous with NEC induction	32 mice (N=8 per group)	not provided	histological NEC incidence/severity<br><br>NEC signs and symptoms<br><br>intestinal inflammation,<br><br>vascular function / hypoxia-ischemia / free radical formation<br><br>intestinal epithelial cell death / proliferation
Maheshwari et al. 2011	Swiss-Webster mice (10-13 days old), NEC induction with formula feeding (200 µL/5 g body weight every 3 hours) and hypoxia (5% O_2 for 2 minutes twice daily before feedings) for 4 days	no enteral TGF-β2	TGF-β2, 100 ng, single dose in the morning for 4 days, simultaneous with NEC induction	36 rats (N=18 per group)	Not provided	histological NEC incidence/severity
Matheson et al. 2014	Sprague-Dawley rats (newborn, 12 hours before scheduled birth), NEC induction with one	formula without rat relaxin	rat relaxin in formula, 0.25 ng/0.1 mL in all feeds, simultaneous with NEC induction	48 rats (N=11-13 per group)	not provided	histological NEC incidence/severity

	time gastric administration of LPS (2 mg/kg), formula feeding (starting 100 µL per feed, increased to 150 µL per feed at 24 hours and to 200 µL per feed at 48 hours, every 4-5 hours) and asphyxia (100% N_2 for 1 minute) and cold exposure (4 °C for 10 minutes) twice daily for 2 days					NEC signs and symptoms<br><br>vascular function / hypoxia-ischemia / free radical formation
Maynard et al. 2010	Sprague-Dawley rats (newborn, 1 day before scheduled birth), NEC induction with formula feeding (total volume 850 µL/day, 6 times a day) and asphyxia (100% N_2 for 1 minute) and cold exposure (4 °C for 10 minutes) twice daily for 4 days	control cow-milk based formula	rat EGF in cow-milk based formula, 500 ng/mL, simultaneous with NEC induction	60 rats (N=24-36 per group)	not provided	intestinal epithelial cell death
Miyake et al. 2019	C57BL/6 mice (5 days old), NEC induction with enteral administration of LPS (4 mg/kg/day), formula feeding (50 µL/g, thrice daily) and hypoxia (5% O_2 for 10 minutes, thrice daily) for 4 days	formula without human breast milk exosomes	human breast milk exosomes from raw milk in formula, simultaneous with NEC induction<br><br>human breast milk exosomes from pasteurized milk in formula, simultaneous with NEC induction	unclear	not provided	histological NEC incidence/severity<br><br>intestinal inflammation,<br><br>intestinal barrier function
Møller et al. 2011	Preterm pigs (92% of gestational age, delivered by caesarian section), NEC induction with parenteral nutrition for the first 2 postnatal days (starting 4 ml/kg/h, after 12h 6 ml/kg/h) and enteral nutrition after 2 days with an oral bolus of 15	deionized water (control for minimal enteral nutrition)<br><br>control formula without supplementatio	bovine OPN, 2.22 g/L, minimal enteral dose of 5 mg/kg body weight pure OPN in sterile deionized water per 3 hours during parenteral nutrition and 2.2 g/L OPN in formula during full enteral nutrition, simultaneous NEC induction	47 pigs (N=5-13 per group)	not provided	histological NEC incidence/severity<br><br>digestion and absorption

	ml/kg formula every 3 hours for 1.5 day	n of OPN, gangliosides or SL	gangliosides enriched bovine milk fraction in formula, 3 g/L resulting in 0.06 g/L gangliosides, simultaneous with start of enteral feeding<br><br>SL enriched bovine milk fraction in formula, 60 g/L resulting in 8.7 g/L SL, simultaneous with start of enteral feeding			
Nguyen et al. 2014	Preterm pigs (92% of gestational age, delivered by caesarian section), NEC induction with parenteral nutrition for the first 2 postnatal days (starting 4 ml/kg/h on day 1, 6 ml/kg/h on day 2) with minimal enteral feeding (3 mL/kg per 3 hours on day 1 and 5 mL/kg per 3 hours on day 2) and full enteral nutrition after 2 days with an oral bolus of 15 ml/kg formula every 3 hours for 2 days	formula without supplementation of bovine lactoferrin	bovine lactoferrin in formula, 10 g/L, during minimal enteral feeding and full enteral feeding, simultaneous with NEC induction	28 pigs (N=13-15 per group)	not provided	histological NEC incidence/severity<br><br>intestinal inflammation,<br><br>intestinal barrier function<br><br>digestion and absorption
Nguyen et al. 2016	Preterm pigs (90-92% of gestational age, delivered by caesarian section), NEC induction with parenteral nutrition for the first 2 postnatal days (starting 4 ml/kg/h on day 1, 6 ml/kg/h on day 2) with minimal enteral feeding (3 mL/kg per 3 hours on day 1 and	formula without supplementation of bovine lactoferrin	bovine lactoferrin in formula, 10 g/L, during minimal enteral feeding and full enteral feeding, simultaneous with NEC induction	28 pigs (N=13-15 per group)	not provided	histological NEC incidence/severity<br><br>intestinal epithelial cell death

	5 mL/kg per 3 hours on day 2) and full enteral nutrition after 2 days with an oral bolus of 15 ml/kg formula every 3 hours for 2 days					
Niño et al. 2017	C57BL/6 mice (7-8 days old), NEC induction with administration of enteric bacteria isolated from an infant with NEC in formula, formula feeding (5 times a day for 4 days) and hypoxia (5% O_2, 95% N_2 for 10 minutes, twice daily) for 4 days	no ATRA	ATRA, dissolved in 1:1 DMSO and corn oil, final concentration 6 mg/mL, 50 μg/mouse, administered daily by gavage, simultaneous with NEC induction	at least N=5 per experimental group	not provided	histological NEC incidence/severity<br><br>intestinal inflammation<br><br>intestinal epithelial cell death / proliferation
Ohtsuka et al. 2011	Sprague-Dawley rats (newborn on day 20 of gestation), NEC induction with one time orogastric administration of formula (150 μL)	control maternal diet (soybean oil)	DHA enriched diet of mother, 49% DHA, 51% soybean oil of total fat, from day 7 to 20 of gestation, prior to NEC induction<br><br>EPA enriched diet of mother, 49% EPA, 51% soybean oil of total fat, from day 7 to 20 of gestation, prior to NEC induction	6 pregnant rats (N=2 per group, resulting in N=20-28 pups per group, 11 control pups)	not provided	histological NEC incidence/severity<br><br>NEC survival<br><br>intestinal inflammation
Olson et al. 2016	Sprague-Dawley rats (newborn on day 21 of gestation), NEC induction with formula feeding (starting 100 μL per feed and advanced to 400 μL on day 4, 5 times a day) and asphyxia (100% N_2 for 90 seconds) followed by cold exposure (4 °C for 10 minutes) thrice daily for 4 days	sterile water	*Lactobacillus reuteri* DSM 20016 in sterile water, $1*10^8$ CFU in 100 μL, once via oral gavage immediately after delivery, simultaneous with NEC induction<br><br>*Lactobacillus reuteri* DSM 20016 grown on unloaded dextranomer microspheres in sterile water, $1*10^8$	168 rats (N=10-48 per group)	not provided	histological NEC incidence/severity<br><br>intestinal barrier function

			CFU in 100 μL, once via oral gavage immediately after delivery, simultaneous with NEC induction<br><br>*Lactobacillus reuteri* DSM 20016 grown on MRS broth loaded dextranomer microspheres in sterile water, 1*10^8 CFU in 100 μL, once via oral gavage immediately after delivery, simultaneous with NEC induction			
Olson et al. 2018	Sprague-Dawley rats (newborn on day 20.5 of gestation), NEC induction with one time intragastric administration of LPS (2 mg/kg), formula feeding (starting 100 μL per feed and advanced to 400 μL on day 4, 5 times a day) and asphyxia (<1.5% O_2 for 90 seconds) followed by cold exposure (4 °C for 10 minutes) thrice daily for 4 days	sterile 0.9% saline	*Lactobacillus reuteri* DSM 20016 in sterile 0.9% saline, 2*10^8 CFU in 100 μL, once via oral gavage immediately after delivery, simultaneous with NEC induction<br><br>*Lactobacillus reuteri* DSM 20016 grown on unloaded Sephadex microspheres in sterile 0.9% saline, 2*10^8 CFU in 100 μL, once via oral gavage immediately after delivery, simultaneous with NEC induction<br><br>*Lactobacillus reuteri* DSM 20016 grown on sucrose-loaded dextranomer microspheres in sterile 0.9% saline, 2*10^8 CFU in 100 μL, once via oral gavage immediately after delivery, simultaneous with NEC induction	279 rats (N=43-50 per group)	not provided	histological NEC incidence/severity<br><br>NEC survival<br><br>intestinal inflammation<br><br>intestinal barrier function<br><br>microbiome alterations

			Lactobacillus reuteri DSM 20016 grown on maltose-loaded Sephadex microspheres in sterile 0.9% saline, 2*10^8 CFU in 100 µL, once via oral gavage immediately after delivery, simultaneous with NEC induction			
Pisano et al. 2020	Sprague-Dawley rats (newborn on day 21 of gestation), NEC induction with one time intragastric administration of LPS (2 mg/kg) with the second feed, formula feeding (starting 100 µL per feed and advanced with 100 µL per day to 400 µL on day 4, every 4 hours), hypoxia (<1.5% O_2 for 90 seconds) thrice daily and cold exposure (4 °C for 10 minutes) thrice daily for 4 days	sterile water intraperitoneal	human breast milk extracellular vesicles in formula, 1*10^8 with every feed, simultaneous with NEC induction	142 rats (N=13-70 per group)	not provided	histological NEC incidence/severity
Quintanilla et al. 2014	Sprague-Dawley rats (newborn), NEC induction with formula feeding (starting 150 µL per feed on day 1, 4 times dialy), hypoxia (5% O_2, 95% N_2 for 10 minutes) thrice daily and cold exposure (4 °C for 10 minutes) thrice daily for 3 days	formula without surfactant protein A	surfactant protein A in formula, 5 µg in 600 µL formula, total dose of 5 µg per animal per day	42 rats (N=6-10 per group)	not provided	histological NEC incidence/severity<br><br>NEC survival<br><br>intestinal inflammation
Radulescu et al. 2009	Sprague-Dawley rats (newborn on day 21.5 of gestation), NEC induction with one time intragastric administration of LPS (2 mg/kg) 8 hours after birth, formula feeding (starting	formula without HB-EGF or EGF	dosing interval experiment: HB-EGF in formula, 800 µg/kg/dose, one / two / three / four or six times a day<br><br>*Escherichia coli* vs *Pichia pastoris*	203 rats (experiment administratio n frequency) 199 rats	not provided	histological NEC incidence/severity<br><br>NEC survival

	100 µL per feed and advanced as tolerated to 400 µL on day 4, every 4 hours), hypoxia (100% N_2 for 1 minute) followed by cold exposure (4 °C for 10 minutes) twice daily for 4 days		derived HB-EGF comparison experiment:<br>HB-EGF derived from *Eschierichia coli* in formula, 600 / 800 or 1000 µg/kg/dose, four or six times a day<br><br>HB-EGF derived from *Pichia pastoris* in formula, 600 / 800 or 1000 µg/kg/dose, four or six times a day<br><br>HB-EGF and EGF comparison experiment:<br>HB-EGF in formula, 800 µg/kg/dose<br>EGF in formula, 800 µg/kg/dose<br>EGF in formula, 570 µg/kg/dose (molarity equivalent to HB-EGF 800 µg/kg/dose)<br><br>prophylactic vs therapeutic HB-EGF administration experiment:<br>HB-EGF in formula, 800 µg/kg/dose, from first feeding 2h after birth or started at 12, 24, 48 or 72h after birth	(dosage experiment)<br><br>120 rats (comparison HB-EGF and EGF)<br><br>137 rats (intervention starting directly after birth or later)		
Ran-Ressler et al. 2011	Sprague-Dawley rats (newborn, 1 day before scheduled birth), NEC induction with formula feeding (total volume 850 µL/day, 6 times a day) and asphyxia (100% N_2 for 1 minute) followed by cold exposure (4 °C	rat formula without supplementation of BCFA	20% w/w BCFA mixture in rat formula, 25% iso-14:0, 20% anteiso-15:0, 25% iso-16:0, 8% anteiso-17:0, 10% iso-18:0 and 12% iso-20:0, simultaneous with NEC induction	73 rats (N=15-35 per group)	Yes, power calculation based on estimated mean and dispersion for NEC scores, α	histological NEC incidence/severity<br><br>NEC signs and symptoms<br><br>intestinal inflammation

	for 10 minutes) twice daily for 4 days				0.05 and β 80%; required sample size 21	microbiome alterations
Rasmussen et al. 2017	Preterm pigs (90-92% of gestational age, delivered by caesarian section), NEC induction with parenteral nutrition for the first 2 postnatal days (4-6 ml/kg/h) with minimal enteral feeding (3 mL/kg per 3 hours) and full enteral nutrition after 2 days with an oral bolus of 15 ml/kg formula every 3 hours for 2 days<br><br>Preterm pigs (90-92% of gestational age, delivered by caesarian section), NEC induction with parenteral nutrition for the first 7 postnatal days (4-6 ml/kg/h) with minimal enteral feeding with slowly increasing amounts (3-17 mL/kg per 3 hours) and full enteral nutrition after 7 days with an oral bolus of 14-17 ml/kg formula every 3 hours for 4 days	control formula without HMO, but with maltodextrin (45-46 g/L)	4 different HMO in formula, 5.0 g/L in most of the experiments, 10 g/L during 4 days' minimal enteral nutrition of the longer study period (0.16-0.64 g/kg/day during these 4 days), the 4 HMO cover the most abundant HMO in milk and represent the characteristic features of naturally occurring HMO, simultaneous with NEC induction<br><br>more than 25 different HMO in formula, 7.0 g/L, more than 25 HMO mimicking the naturally occurring HMO in human milk), simultaneous with NEC induction	112 pigs (N=14-23 per group)	not provided	histological NEC incidence/severity<br><br>NEC signs and symptoms<br><br>intestinal inflammation<br><br>intestinal barrier function<br><br>intestinal epithelial proliferation<br><br>microbiome alterations<br><br>digestion and absorption
Ren et al. 2019	Preterm pigs (89-92% of gestational age, delivered by caesarian section), IA LPS from	formula without supplemented OPN or CGMP	OPN in formula, 2.2 g/L, simultaneous with NEC induction	44 pigs (N=10-12 per group)	not provided	histological NEC incidence/severity

	E. Coli 055:B5 1 mg in an area close to the mouth at 103 days of gestational age, NEC induction by parenteral nutrition (amount gradually decreasing from 96/ml/kg/day to 48 ml/kg/day) and enteral nutrition (gradually increasing from 24 ml/kg/day to 120 ml/kg/day) for 5 days		CGMP in formula, 30 g/L, simultaneous with NEC induction			intestinal inflammation,<br><br>intestinal barrier function
Rentea et al. doi: 10.1016/j.jpedsurg.2012.03.018 2012	Sprague-Dawley rats (newborn, 1 day before scheduled birth), NEC induction with enteral administration of LPS (2 mg/kg LPS) per feed, formula feeding and hypoxia (5% O_2, 95% N_2 for 10 minutes thrice daily) for 4 days	formula without supplemented IAP	IAP in formula, 0.4 U/kg, once daily, simultaneous with NEC induction<br><br>IAP in formula, 4 U/kg, once daily, simultaneous with NEC induction<br><br>IAP in formula, 40 U/kg, once daily, simultaneous with NEC induction	N=10-31 per group	not provided	histological NEC incidence/severity<br><br>intestinal barrier function
Rentea et al. doi: 10.1016/j.jss.2012.05.039 2012	Sprague-Dawley rats (newborn, 1 day before scheduled birth), NEC induction with enteral administration of LPS (2 mg/kg LPS) per feed, formula feeding every 4 hours and hypoxia (5% O_2, 95% N_2 for 10 minutes thrice daily) for 3 days	formula without supplemented IAP	IAP in formula, 0.4 U/kg, once daily, simultaneous with NEC induction<br><br>IAP in formula, 4 U/kg, once daily, simultaneous with NEC induction<br><br>IAP in formula, 40 U/kg, once daily, simultaneous with NEC induction	N=7-17 per group	not provided	systemic inflammation
Rentea et al. 2013	Sprague-Dawley rats (newborn, 1 day before scheduled birth), NEC induction with enteral administration of LPS (2 mg/kg LPS) per feed, formula feeding every 4 hours and hypoxia (5%	formula without supplemented IAP	IAP in formula, 0.4 U/kg, once daily, simultaneous with NEC induction<br><br>IAP in formula, 4 U/kg, once daily, simultaneous with NEC induction	at least N=7 per group	not provided	intestinal inflammation<br><br>intestinal barrier function

	O_2, 95% N_2 for 10 minutes) thrice daily for 1 day		IAP in formula, 40 U/kg, once daily, simultaneous with NEC induction			vascular function / hypoxia-ischemia / free radical formation
Rudloff et al. 2019	Preterm pigs (90-92% of gestational age, delivered by caesarian section), NEC induction with parenteral nutrition for the first 2 postnatal days (4-6 ml/kg/h) with minimal enteral feeding (3 mL/kg per 3 hours) and full enteral nutrition after 2 days with an oral bolus of 15 ml/kg formula every 3 hours for 2 days<br><br>Preterm pigs (90-92% of gestational age, delivered by caesarian section), NEC induction with parenteral nutrition for the first 7 postnatal days (4-6 ml/kg/h) with minimal enteral feeding with slowly increasing amounts (3-17 mL/kg per 3 hours) and full enteral nutrition after 7 days with an oral bolus of 14-17 ml/kg formula every 3 hours for 4 days	control formula without HMO, but with maltodextrin (45-46 g/L)	4 different HMO in formula, 5.0 g/L in most of the experiments, 10 g/L during 4 days' minimal enteral nutrition of the longer study period (0.16-0.64 g/kg/day during these 4 days), the 4 HMO cover the most abundant HMO in milk and represent the characteristic features of naturally occurring HMO, simultaneous with NEC induction<br><br>more than 25 different HMO in formula, 7.0 g/L, more than 25 HMO mimicking the naturally occurring HMO in human milk), simultaneous with NEC induction	112 pigs (N=30-44 per group)	not provided	microbiome alterations
Satoh et al. 2016	Sprague-Dawley rats (newborn on day 21 of gestation), NEC induction with formula feeding thrice daily and hypoxia (5% O_2,	formula without *Bifidobacterium breve* M-16V	*Bifidobacterium breve* M-16V in formula, 6*10^7 CFU per day	60 rats (N=17-23 per group)	not provided	histological NEC incidence/severity

	95% N_2 for 10 minutes) thrice daily before each feeding for 4 days					NEC survival<br><br>intestinal inflammation<br><br>intestinal barrier function<br><br>intestinal epithelial cell death
Shen et al. 2019	Sprague-Dawley rats (newborn), NEC induction with formula feeding (200 µL every 4 hours on the first day, increasing with 50 µL in the subsequent day) and asphyxia (100% N_2 for 1.5 minute) followed by cold stress (4 °C for 10 minutes) twice daily for 4 days	formula without supplemented lactadherin	lactadherin in formula, 10 ug/g/day, simultaneous with NEC induction	45 rats (N=15 per group)	not provided	histological NEC incidence/severity<br><br>NEC signs and symptoms<br><br>NEC survival<br><br>intestinal barrier function
Sheng et al. 2014	Sprague-Dawley rats (newborn on day 21.5 of gestation), NEC induction with formula feeding (100 µL every 4h and increased to 300-400 µL if tolerated) and asphyxia (100% N_2 for 60 seconds) followed by cold exposure (4°C for 10 minutes) twice daily for 4 days	saline	human β defensin, 100 µg/kg in 100 µL, daily before asphyxia, simultaneous with NEC induction	68 rats (N=12-24 per group)	not provided	histological NEC incidence/severity<br><br>NEC signs and symptoms<br><br>NEC survival<br><br>intestinal inflammation

						systemic inflammation<br><br>intestinal barrier function
Shiou et al. 2011	Sprague-Dawley rats (newborn on day 20 of gestation), NEC induction with formula feeding (100 µL every 3 hours, incrementally increased to 250 µL) and hypoxia (5% O_2, 95% N_2 for 10 minutes, thrice daily) for 5 days	formula without supplemented EPO or TGF-ß	EPO in formula, 0.1 unit/mL, simultaneous with NEC induction<br><br>TGF-β in formula, 30 ng/ml, simultaneous with NEC induction	132 rats (N=20-56 per group)	not provided	histological NEC incidence/severity<br><br>intestinal barrier function
Shiou et al. 2013	Sprague-Dawley rats (newborn on day 20 of gestation), NEC induction with 10^7 CFU of both Serratia marcescens, Klebsiella pneumonia and Streptococcus viridans, formula feeding every 3 hours and hypoxia (5% O_2, 95% N_2 for 10 minutes, thrice daily) for 5 days	vehicle	TGF-β1 in formula, 30 ng/ml, simultaneous with NEC induction	116 rats (N-20-48 per group)	not provided	histological NEC incidence/severity<br><br>intestinal inflammation<br><br>systemic inflammation
Siggers et al. 2008	Preterm pigs (107d of gestational age, delivered by caesarian section), NEC induction with parenteral nutrition for the first 36 postnatal hours (starting 4 ml/kg/h, after 12h 6 ml/kg/h) and full enteral nutrition after 36 hours with an oral bolus of 15 ml/kg formula every 3 hours for 2 days	peptone water placebo	probiotic mixture in 1% peptone water, *Bifidobacterium animalis* DSM15954, *Lactobacillus acidophilus* DSM13241, *Lactobacillus casei* ATCC55544, *Lactobacillus pentosus* DSM14025 and *Lactobacillus plantarum* DSM13367 all strains 10^9 CFU/gram viable lypophilzed bacteria, total concentration of $5*10^9$ CFU/3 mL peptone water, 2 mL/kg birth weight administered every 6	28 pigs (N=5-13 per group)	not provided	histological NEC incidence/severity<br><br>microbiome alterations<br><br>digestion and absorption

			hours during period of parenteral nutrition and every 3 hours during period of enteral nutrition			
Siggers et al. 2013	Preterm pigs (92% of gestational age, delivered by caesarian section), NEC induction with parenteral nutrition for the first 2 postnatal days (starting 4 ml/kg/h, after 12h 6 ml/kg/h) and full enteral nutrition after 2 days with an oral bolus of 15 ml/kg formula every 3 hours for 2 days<br><br>Preterm pigs (92% of gestational age, delivered by caesarian section), NEC induction with exclusive parenteral nutrition for the first 2 postnatal days (starting 4 ml/kg/h, after 12h 6 ml/kg/h) and declining parenteral feeding and gradually increasing enteral nutrition after 2 days with an oral bolus of formula every 3 hours reaching 15 ml/kg after 9 hours for 2 days	formula without porcine amniotic fluid	porcine amniotic fluid, 10 ml/kg pure bolus during parenteral nutrition every 3 hours and as water fraction (80%) in 15 ml/kg formula during full enteral nutrition, simultaneous with NEC induction<br><br>porcine amniotic fluid, as water fraction (80%) in 15 ml/kg formula during full enteral nutrition, on postnatal day 3-4	30 pigs (N=7-13 per group)	not provided	histological NEC incidence/severity<br><br>NEC signs and symptoms<br><br>intestinal inflammation<br><br>vascular function / hypoxia-ischemia / free radical formation<br><br>microbiome alterations<br><br>digestion and absorption
Sodhi et al. 2018	C57BL/6J mice (7-8 days old), NEC induction with supplementation of enteric bacteria isolated from an infant with severe NEC (12.5 µL stool slurry in 1 mL formula) in	control formula, 100% TAG-rich oils; mixture of 39% high oleic safflower oil, 29% soya oil	pre-digested formula, 50% TAG-rich oils; 17.5% soybean NEFA, 20% 2-monoacylglycerol palmitate, 10.3% phospholipid lecithin, 34.8% high oleic safflower oil, 14.8% coconut oil, 135 mg/L DHA, 322	at least N=8 per group	not provided	histological NEC incidence/severity<br><br>NEC signs and symptoms

	formula feeding (50 μL/g, 5 times a day) and hypoxia (5% O_2 95% N_2 for 10 minutes twice daily) for 4 days	and 27.9% coconut oil; 146 mg/L DHA, 312 mg/L AA acid	mg/L AA, simultaneous with NEC induction<br><br>very low fat formula, 161 mg/L DHA, 355 mg/L AA, fat replaced by lactose, simultaneous with NEC induction			intestinal inflammation<br><br>vascular function / hypoxia-ischemia / free radical formation
Sodhi et al. 2020	C57BL/6J mice (7-8 days old), NEC induction with supplementation of enteric bacteria isolated from an infant with severe NEC (12.5 μL stool slurry in 1 mL formula) in formula feeding (50 μL/g, 5 times a day) and hypoxia (5% O_2, 95% N_2 for 10 minutes twice daily) immediately after feed for 4 days	formula without supplemented 2′-FL, 6′-SL, lactose or GOS	2′-FL in formula, 10 mg/kg, 200 μL/mouse, simultaneous with NEC induction<br><br>6′-SL in formula, 10 mg/kg, 200 μL/mouse, simultaneous with NEC induction<br><br>lactose in formula, 10 mg/kg, 200 μL/mouse, simultaneous with NEC induction<br><br>GOS in formula, 10 mg/kg, 200 μL/mouse, simultaneous with NEC induction	at least N= 5 per group	not provided	histological NEC incidence/severity<br><br>NEC signs and symptoms<br><br>intestinal inflammation<br><br>vascular function / hypoxia-ischemia / free radical formation,<br><br>intestinal epithelial cell death
Sodhi et al. 2020	Yorkshire piglets (~90% gestation, caesarian delivery), NEC with supplementation of enteric bacteria isolated from an infant with surgical NEC (12.5 μL stool slurry in 1 mL formula) in formula feeding (15 mL/kg every 3 hours) for 4 days	formula without supplemented 2′-FL or 6′-SL	2′-FL in formula, 10 mg/ml, simultaneous with NEC induction<br><br>6′-SL in formula, 10 mg/ml, simultaneous with NEC induction<br><br>2′-FL + 6′-SL in formula, 5 mg/ml	at least N= 4 per group	not provided	histological NEC incidence/severity<br><br>NEC signs and symptoms<br><br>intestinal inflammation

			each, simultaneous with NEC induction			vascular function / hypoxia-ischemia / free radical formation,<br><br>intestinal epithelial cell death
Su et al. 2013	Friend Virus B-Type mice (newborn on day 18.5 of gestation), NEC induction with formula feeding (30 μL every 3 hours and increased to a maximum of 50 μL per feed at day 4) and asphyxia (100% N_2, for 1 minute) and cold exposure (4 °C for 10 minutes) once daily for 4 days	formula without HB-EGF	HB-EGF in formula, 800 μg/kg/dose, added to each feed, simultaneous with NEC induction	182 mice (N=15-67 per group)	not provided	histological NEC incidence/severity<br><br>intestinal barrier function<br><br>intestinal epithelial proliferation
Tang et al. 2019<br><br>article in Chinese	C57BL/6J mice (10 day old), NEC induction with formula feeding (300~500 μL/g, 5 times a day) and hypoxia (N_2 12 L/min for 1.5 minute) and cold exposure (4 °C for 10 minutes) thrice daily for 3 days	PBS	*Lactobacillus reuteri* DSM17938, 10^6 CFU/g/day dissolved in PBS at 10^7 CFU/mL, intragastric once every day, simultaneous with NEC induction	96 mice (N=32 per group)	not provided	histological NEC incidence/severity<br><br>NEC signs and symptoms<br><br>intestinal inflammation<br><br>vascular function / hypoxia-ischemia / free radical formation

Tian et al. 2017	Sprague-Dawley rats (3 days old), NEC induction with formula feeding (150 μL 4 times a day on day 1 and increased 100 μL per feed per day) and hypoxia (100% N_2 for 1.5 minute) followed by cold exposure (4 °C for 10 minutes) thrice daily for 3 days	Formula without supplemented IGF1	IGF1 in formula, 22 mg/L, simultaneous with NEC induction	60 rats (N=20 per group)	not provided	histological NEC incidence/severity<br><br>NEC signs and symptoms,<br><br>intestinal inflammation<br><br>intestinal barrier function
Wang et al. doi: 10.1002/mnfr.201900262 2019	C57BL/6 mice (7 days old), NEC induction with enteral LPS (4 mg/kg/day) on postnatal day 7 (part of the animals), formula feeding (100-200 μL every 8 hours) and hypoxia (5% O_2, 95% N_2 for 10 minutes) followed by cold exposure (4 °C for 10 minutes) thrice daily for 3 days	formula without supplemented HMO	HMO in formula, 20 g/L, simultaneous with NEC induction	90 mice (N=13-15 per group)	not provided	histological NEC incidence/severity<br><br>NEC survival<br><br>intestinal inflammation<br><br>systemic inflammation<br><br>intestinal epithelial proliferation
Wang et al. doi: 10.1002/mnfr.201801247 2019	Sprague-Dawley rats (10 days old), NEC induction with formula feeding (50 μL/g body weight 3 times per day) and hypoxia (5% O_2, 95% N_2 for 5 minutes twice daily) for 4 days	formula without supplemented human breast milk exosomes	human breast milk exosomes in formula, 200 μg/mL, isolated from lactating mothers who had delivered preterm infants (24-36 weeks), simultaneous with NEC induction	unclear	not provided	histological NEC incidence/severity<br><br>intestinal epithelial cell proliferation

Wang et al. 2020	C57BL/6 mice (7 days old), NEC induction with formula feeding (100-200 µL every 8 hours) and hypoxia (5% O_2, 95% N_2 for 10 minutes) followed by cold exposure (4 °C for 10 minutes) thrice daily for 3 days	formula without supplemented HMO	HMO in formula, 5 g/L, simultaneous with NEC induction<br>HMO in formula, 10 g/L, simultaneous with NEC induction<br>HMO in formula, 20 g/L, simultaneous with NEC induction	70 mice (N=10-15 per group)	not provided	histological NEC incidence/severity<br><br>NEC survival<br><br>intestinal inflammation<br><br>systemic inflammation<br><br>intestinal epithelial cell death / proliferation
Wei et al. doi:10.1038/pr.2015.63 2015	C57BL/6 mice (newborn), NEC induction with formula feeding (300 µL every 3 hours on day 1 and increased to a maximum of 500 µL per feed on day 4) and asphyxia (100% N_2 for 1 minute) followed by cold exposure (4 °C for 10 minutes) twice daily for 4 days	formula without supplemented HB-EGF	HB-EGF in formula, 800 µg/kg per dose in each feed, simultaneous with NEC induction	246 mice (N=22-49 per group)	not provided	intestinal barrier function<br><br>enteric nervous system
Wei et al. doi: 10.1016/j.jss.2015.03.023 2015	C57BL/6 mice (newborn), NEC induction with formula feeding and hypoxia (5% O_2, 95% N_2 for 1 minute) followed by cold exposure (4 °C for 10 minutes) twice daily for 4 days	formula without supplemented HB-EGF	HB-EGF in formula, 800 µg/kg per dose in each feed, simultaneous with NEC induction	92 mice (N=17-48 per group)	not provided	histological NEC incidence/severity<br><br>intestinal inflammation
Whitehouse et al. 2010	Sprague-Dawley rats (newborn), NEC induction with formula feeding (200 µL thrice daily)	formula without supplemented bovine calf IAP	bovine calf IAP in formula, four glycine units in the morning feed, simultaneous wit NEC induction	89 rats (N=13-28 per group)	not provided	histological NEC incidence/severity

	supplemented with LPS (2 mg/kg per feed, all or part of the feeds) and hypoxia (5% O_2 for 10 minutes thrice daily) for 4 days					
Wu et al. 2017	Sprague-Dawley rats (newborn on day 20-21 of gestation), NEC induction with formula feeding (150 μL every 4 hours and increased to a maximum of 200 μL per feed after 1 day if tolerated) and asphyxia (100% N_2 for 1.5 minute) followed by cold exposure (4 °C for 10 minutes) twice daily for 3 days	formula without bacterial strain	*Bifidobacterium adolescentis* in formula, 1.0*10[8] per day	45 rats (N=14-16 per group)	not provided	histological NEC incidence/severity<br><br>NEC signs and symptoms<br><br>NEC survival<br><br>intestinal inflammation
Wu et al. 2019	C57BL/6 mice (5 days old), NEC induction with enteral administration of LPS (4 mg/kg/day) on postnatal day 6-7, formula feeding (50 μL/g, thrice daily) and hypoxia (5% O_2 for 10 minutes, thrice daily) for 5 days	lactose in formula, 2 mg/g/day	HMO from pooled mature breast milk in formula, 20 mg/mL (2% weight/volume), daily HMO intake 2-3 mg/g body weight, simultaneous with NEC induction<br><br>some experiments rutin hydrate, 0.5 mg/g body weight/day (proteindisulfide isomerase antagonist) added to formula, simultaneous with NEC induction	at least N=6 per group	not provided	histological NEC incidence/severity<br><br>NEC signs and symptoms<br><br>intestinal barrier function
Xiao et al. 2018	C57BL/6J mice (8 days old), NEC induction with formula feeding (every 4 hours) and asphyxia (100% N_2 for 1 minute) followed by cold exposure (4 °C for 10 minutes) twice daily for 3 days	PBS	vitamin A, 20 IU, intragastric once daily from postnatal day 1-7, prior to NEC induction	24 mice (N=12 per group)	not provided	histological NEC incidence/severity<br><br>intestinal inflammation,<br><br>intestinal barrier function

						microbiome alterations
Xu et al. 2013	Sprague-Dawley rats (newborn), NEC induction with formula feeding (100 μL every 4 hours and increased if tolerated by 50 μL every 3 feeds to a maximum of 400 μL per feed on day 4) and asphyxia (100% N_2 for 1 minute) followed by cold exposure (4 °C for 10 minutes) twice daily for 4 days	formula without supplemented GD3	GD3 in formula, 15 μg/mL, simultaneous with NEC induction	90 rats (N=30 per group)	not provided	histological NEC incidence/severity<br><br>NEC signs and symptoms<br><br>intestinal inflammation
Yakut et al. 2020	Wister rats (newborn), NEC induction with one time intraperitoneal LPS (1 mg/kg in distilled water from E. coli O111:B4) on the first day, formula feeding (starting 200 μL every 3 hours and daily increased by 100 μL if tolerated) and asphyxia (100 CO_2 for 10 minutes), hyperoxia (97% O_2 for 5 minutes) and cold exposure (4 °C for 5 minutes) twice daily for 3 days	distilled water	Foeniculum vulgare extract in distilled water, 200 mg/kg/day, 0.8 ml/kg/day, oral once daily, simultaneous with NEC induction	42 rats (N=12 per group)	not provided	histological NEC incidence/severity<br><br>NEC signs and symptoms<br><br>intestinal inflammation,<br><br>vascular function / hypoxia-ischemia / free radical formation<br><br>intestinal epithelial cell death
Yang et al. 2012	Sprague-Dawley rats (newborn on day 21.5 of gestation), NEC induction with formula feeding	Formula without	HB-EGF in formula, 800 μg/kg per dose in each feed, simultaneous with NEC induction	197 rats (N=10-43 per group)	not provided	histological NEC incidence/severity

	(starting 100 μL every 4 hours and increased to a maximum of 400 μL per feed on day 4) and asphyxia (100% N_2 for 1 minute) followed by cold stress (4 °C for 10 minutes) twice daily for 4 days	supplemented HB-EGF				NEC signs and symptoms<br><br>NEC survival<br><br>intestinal barrier function
Yang et al. 2020	Sprague-Dawley rats (2 days old), NEC induction with enteral LPS (5 mg/mL) from day 3 of experiment (5 days old) and formula feeding (starting 200 μL every 4 hours and increased to 300 μL per feed) for 8 days	formula without supplementation of glutamine or MPLs	glutamine in formula, 200 mg/kg, simultaneous with NEC induction<br><br>MPLs in formula, 50 mg/kg, simultaneous with NEC induction<br><br>MPLs in formula, 100 mg/kg, simultaneous with NEC induction<br><br>MPLs in formula, 200 mg/kg, simultaneous with NEC induction	72 rats (N=12 per group)	not provided	histological NEC incidence/severity<br><br>NEC signs and symptoms<br><br>intestinal inflammation<br><br>intestinal epithelial cell death
Yin et al. 2020	Rats (newborn), NEC induction with hypoxia (5% O_2 for 10 minutes) and hypothermia (8 °C for 10 minutes)	1% gum acacia solution	curcumin dissolved in 1% gum acacia solution, 20 mg/kg (low dose), orally administered<br><br>curcumin dissolved in 1% gum acacia solution, 50 mg/kg (high dose), orally administered	20 rats	not provided	histological NEC incidence/severity intestinal inflammation,<br><br>intestinal epithelial cell death
Yu et al. 2009	Sprague-Dawley rats (newborn on day 21 of gestation), NEC induction with one time intragastric LPS (2 mg/kg) 8 hours after birth, formula feeding (100 μL every 4 hours	formula without supplemented HB-EGF	HB-EGF in formula, 800 μg/kg per dose in each feed, simultaneous with NEC induction	128 rats (N=63-65 per group)	not provided	histological NEC incidence/severity<br><br>vascular function / hypoxia-

	and increased to a maximum of 400 μL per feed) and asphyxia (100% N_2, for 1 minute) followed by cold exposure (4 °C for 10 minutes) twice daily for 1,2 or 3 days					ischemia / free radical formation
Yu et al. 2013	Sprague-Dawley rats (newborn, 1 day before scheduled birth), NEC induction with 10^7 CFU of both Serratia marcescens, Klebsiella pneumonia and Streptococcus viridans in 100 μL formula once daily, formula feeding (100 μL every 3 hours, incrementally increased to 250 μL) and hypoxia (5% O_2, 95% N_2 for 10 minutes thrice daily) for 5 days	Formula without supplemented EPO	EPO in formula, 0.1 μg/mL, simultaneous with NEC induction	at least N=3 per group	not provided	intestinal epithelial cell death
Yu et al. 2014	Sprague-Dawley rats (newborn), NEC induction with formula feeding (200 μL twice daily) and hypoxia (5% O_2, 95% N_2 for 10 minutes) thrice daily for 4 days	formula without added HMO, GOS or synthetic disialyl glycans	HMO in formula, 2 mg/mL, simultaneous with NEC induction<br><br>GOS in formula, 2 mg/mL, simultaneous with NEC induction<br><br>DSLNnT (synthetic disialyl glycan) in formula, 300 mg/ml, simultaneous with NEC induction<br><br>3′′′-sLNnT (synthetic monosialyl glycan) in formula, 300 mg/ml, simultaneous with NEC induction	174 rats (N=11-33 per group)	not provided	histological NEC incidence/severity

			DS'LNT (synthetic disialyl glycan) in formula, 300 mg/ml, simultaneous with NEC induction<br><br>GD3 (synthetic disialyl glycan) in formula, 300 mg/ml, simultaneous with NEC induction<br><br>DSLac (synthetic disialyl glycan) in formula, 300 mg/ml, simultaneous with NEC induction			
Yu et al. 2017	Sprague-Dawley rats (newborn), NEC induction with formula feeding (200 μL twice daily) and hypoxia (5% O_2, 95% N_2 for 10 minutes) thrice daily for 4 days	formula without added HMO or synthetic disialyl glycans	HMO in formula, 10 mg/mL, simultaneous with NEC induction<br><br>Neu5Gc-DS'LNT (synthetic disialyl glycan) in formula, 300 μM, simultaneous with NEC induction<br><br>DS'LNnT (synthetic disialyl glycan) in formula, 300 μM, simultaneous with NEC induction<br><br>DSTa (synthetic disialyl glycan) in formula, 300 μM, simultaneous with NEC induction<br><br>DSGalB (synthetic disialyl glycan) in formula, 300 μM, simultaneous with NEC induction<br><br>DSLNnT (synthetic disialyl glycan) in formula, 300 μM, simultaneous with NEC induction	139 rats (N=9-22 per group)	not provided	histological NEC incidence/severity

			DS'LNT (synthetic disialyl glycan) in formula, 300 μM, simultaneous with NEC induction			
Zhang et al. 2019	Sprague-Dawley rats (newborn), NEC induction with formula feeding (150 μL every 4 hours, incrementally increased to 300-400 μL per feed) and hypoxia (5% O_2, 95% N_2 for 1.5 minute) followed by cold exposure (4 °C for 10 minutes) twice daily for 4 days	formula without supplemented MFGM	MFGM in formula, 6 g/L, simultaneous with NEC induction<br><br>MFGM in formula, 12 g/L, simultaneous with NEC induction	62 rats (N=12-20 per group)	not provided	histological NEC incidence/severity<br><br>NEC signs and symptoms<br><br>NEC survival<br><br>intestinal inflammation<br><br>vascular function / hypoxia-ischemia / free radical formation
Zhou et al. 2014	Sprague-Dawley rats (newborn on day 21 of gestation), NEC induction with formula feeding (starting 150 μL per feed every 4 hours, after 24h 200μL per feed), followed by asphyxia (N_2 flow 10 L/minute, after reaching 0% O2 for 1.5 minute) and cold stress (4 °C for 10 minutes) twice daily for 3 days	formula without supplemented glutamine	glutamine in formula, 0.3g/kg, simultaneous with NEC induction	60 rats (N=20 per group)	not provided	histological NEC incidence/severity intestinal inflammation<br><br>intestinal epithelial cell death
Zhou et al. 2015	Sprague-Dawley rats (newborn), NEC induction with intragastric LPS (30 mg/kg in sterile water)	formula without Bifidobacterium microcapsules	Bifidobacterium microcapsules in formula, $1*10^{10}$ CFU/ml, once daily, simultaneous with NEC induction	75 rats (N=15 per group)	not provided	histological NEC incidence/severity

	once daily and formula feeding (starting 100 μL per feed every 4 hours and increased with 50 μL every 12 hours with a maximum of 300 μL per feed) for 3 days					intestinal inflammation
Zhou et al. 2017	Sprague-Dawley rats (newborn on day 21 of gestation), NEC induction with one time intragastric LPS (2 mg/kg) 8 hours after birth, formula feeding and asphyxia (100% N_2 for 1 minute) followed by cold exposure (4 °C for 10 minutes) twice daily for 4 days	formula without supplemented HB-EGF	HB-EGF in formula, 800 μg/kg per dose in each feed, simultaneous with NEC induction	at least N=3 per group	not provided	enteric nervous system alterations
Zhu et al. 2020	Sprague-Dawley rats (1 day old), NEC induction enteral administration of LPS (10 mg/kg) once daily, formula feeding (400 μL 4 times a day) and hypoxia (N_2 flow 15 L/minute, after reaching 0% O_2 for 1.5 minute) followed by cold exposure (4 °C for 10 minutes) twice daily for 3 days	no fish oil	fish oil, 35% DHA and EPA in total, 0.6 mL/100g/day, once daily for 7 days prior to NEC induction	96 rats (N=32 per group)	not provided	histological NEC incidence/severity<br><br>NEC signs and symptoms<br><br>intestinal inflammation<br>intestinal epithelial cell death

AA: arachidonic acid; AMP, adenosine monophosphate; ATRA: all-trans retinoic acid; BCFA: branched chain fatty acids; CFU, colony forming units; CGMP: caseinoglycomacropeptide; CMP, Cytidine monophosphate; DHA: docosahexaenoic acid; DMSO, Dimethylsulfoxide; DSGalB, disialyl galactobiose; DSLNT, disialyllacto-N-tetraose; DSLNnT, disialyllacto-N-neotetraose; DS'LNT, a2–6-linked disialyllacto-N-tetraose; DS'LNnT, a2–6-linked disialyllacto-N-neotetraose; DSLac, disialyllactose; DSTa, disialyl T-antigen tetraose; 3'''-sLNnt, 3'''-sialyllacto-N-neotetraose; EGF: epidermal growth factor; EPA: eicosapentaenoic acid; EPO: erythropoietin; 2'-FL: 2'-fucosyllactose; FOS, fructo-oligosaccharides; G-CSF: granulocyte colony-stimulating factor; GD3: ganglioside D3; GMP, guanosine monophosphate; GOS, galacto-olgosaccharides; HB-EGF: hemoglobin-binding EGF-like growth factor; HGF, hepatocyte growth factor; HMO: human milk oligosaccharides; IAP: intestinal alkaline phosphatase; IGF1: insulin-like growth factor 1; IMP, inosine monophosphate; LC-FOS: long chain fructo-oligosaccharides; MFGM: milk fat globule membrane; MPL: milk polar lipids; NEC: necrotizing enterocolitis; NEFA, non-esterified fatty acid; OPN: osteopontin; PAF-AH, platelet activating factor acetylhydrolase; PUFA: polyunsaturated fatty acids; SC-GOS: short chain galacto-oligosaccharides; SL: sialic acids; 6'-SL: 6'-sialyllactose; TAG, Triacylglycerol; TGF-β: transforming growth factor β; UMP, uridine monophosphate;

Supplementary table S6. Overview included clinical trials on enteral feeding interventions for prevention of NEC in human infants

Author and year	Type of study and sample size	In- and exclusion criteria	Control	Intervention	Sample size / power calculation	Primary / secondary outcome measures
Akin et al. 2014	single center RCT N=50 neonates	Inclusion: in hospital born, birth weight <1500 g or gestational age <32 weeks at Ankara University School for Medicine between December 2009 and January 2011<br><br>Exclusion: lack of parental consent, severe congenital anomalies, severe perinatal asphyxia, expiring before allocation to treatment group in the first 72h	2 mL saline once a day throughout the hospitalization period after the baby reached 20 mL/kg/feeding/day feeding volume (N=25 neonates)	bovine lactoferrin, 200 mg/day throughout the hospitalization period after the baby reached 20 mL/kg/feeding/day feeding volume (N=25 neonates)	not provided	Primary outcome(s): tolerability and effectiveness of bovine lactoferrin 200 mg/day in the prevention of nosocomial sepsis, NEC and mortality<br><br>Secondary outcome(s): effect of maturation and bovine lactoferrin on Treg levels
Collins et al. 2017	multi center RCT N=1205 neonates	Inclusion: infants <29 weeks that started on enteral feeding in the previous 3 days at one of 13 centers in Australia, New Zealand and Singapore from June 18th 2012 to September 30th 2015.	soy emulsion emulsified with 1% lecithin and containing 20% total fat and 6% protein<br><br>0.5 mL of the soy emulsion contained 55 mg of linoleic acid, 5.7 mg of	DHA emulsion with a microencapsulated aqueous emulsion of fractionated tuna oil (70% of total oil as DHA in triglyceride form); emulsified 1% lecithin and containing 20% total fat and 6% protein	Yes, power calculation based on estimated absolute effect of 10 percent point and a 19% relative difference in incidence of bronchopulmonary dysplasia, power 90%; α 0.05;	Primary outcome(s): incidence of physiological bronchopulmonary dysplasia<br><br>Secondary outcome(s): secondary respiratory outcomes, retinopathy of prematurity, intraventricular

		Exclusion: lack of parental consent, major congenital or chromosomal anomalies, participating in another trial of fatty acid supplementation or receiving intravenous lipids containing fish oil or receiving breastmilk from a mother taking DHA supplements at a dose of more than 250 mg per day	alpha-linolenic acid, 4.8 mg vitamin C and 0.01 mg vitamin E<br><br>emulsion was enterally administered at dose of 0.17 ml/kg/body weight three times daily (total 0.5 ml/kg bodyweight/day) immediately before feeding via a nasogastric or orgastric tube (N=613 neonates)	0.5 mL of the DHA emulsion contained 60 mg of DHA, 4 mg of EPA, 4.6 mg vitamin C, 0.05 mg vitamin E<br><br>emulsion was enterally administered at dose of 0.17 ml/kg/body weight three times daily (total 0.5 ml/kg bodyweight/day) immediately before feeding via a nasogastric or orgastric tube (N=592 neonates)	required sample size 1244 (622 per group)	hemorrhage, sepsis, NEC and measures of safety and tolerance
El-Ganzoury et al. 2014	single center RCT N=90 neonates	Inclusion: gestational age ≤ 33 weeks at the NICU of Ain-Shams University hospitals between March 2013 and March 2014.<br><br>Exclusion: lack of parental consent, congenital or acquired anomalies of the gastrointestinal tract and previous use of cytokines or intravenous immunoglobulin.	1 mL of distilled water once daily (N=30 neonates	recombinant EPO, single enteral daily dose of 80 IU/kg (N=20 neonates)<br><br>recombinant G-CSF, single enteral daily dose of 4.5 μg/kg (N=20 neonates)<br><br>recombinant EPO, single enteral daily dose of 80 IU/kg, and recombinant G-CSF, single enteral	Yes, power calculation based on mean total time to full enteral feeding of 35 +/- 5 days, , estimated 30% relative difference time to full enteral feeding, power 80%; α 0.05; required sample size 20	Primary outcome(s): time to full enteral feeding<br><br>Secondary outcome(s): weight gain, NEC incidence, NEC related death, length of hospital stay, hospital readmission, adverse effects of treatment

				daily dose of 4.5 μg/kg (N=20 neonates)<br><br>all interventions started on the day of start of enteral feeding and continued until enteral intake reached 100 ml/kg/day or after a maximum of 7 days		
Hosseini el al. 2019	single center RCT N=150 neonates	Inclusion: gestational age ≤ 28 weeks, birth weight <1250, appropriate for gestational age between June 21st 2016 and February 19th 2018.<br><br>Exclusion: lack of parental consent, severe birth asphyxia, chromosome anomalies, congenital heart disease, congenital intestinal obstruction, omphalocele, gastroschisis, nil per os for more than 3 weeks	routine feeding without any administration (N=50 neonates)	synthetic amniotic fluid (containing sodium chloride, sodium acetate, potassium chloride and G-CSF), 5 ml/kg/day (N=50 neonates)<br><br>recombinant human EPO dissolved in synthetic amniotic fluid (containing sodium chloride, sodium acetate, potassium chloride and G-CSF) 4400 mμ/ml (N=50 neonates)<br><br>all interventions started 3 days after birth and were continued for 21 days	not provided	Primary/secondary outcome(s): gastric residual volume, vomiting, NEC (stage ≥2), NEC requiring surgery, retinopathy of prematurity (stage 2 or 3), intraventricular hemorrhage (grade ≥2), anemia of prematurity, late onset sepsis, mortality

Indrio et al. 2009	single center RCT N=49 neonates	Inclusion: healthy preterm neonates, appropriate for gestational age and normal Apgar score<br><br>Exclusion: lack of parental consent, respiratory distress, congenital malformation, inborn errors of metabolism, proven sepsis or infection	indistinguishable placebo formulation for 30 days (N=12 neonates)<br><br>breastmilk fed control group (N=17 neonates)	prebiotic supplemented formula (0.8 g/dL of a mixture from SC-GOS and LC-FOS 9:1) for 30 days (N=10 neonates)<br><br>probiotic supplemented formula (Lactobacillus reuteri, 10^8 CFU per day delivered in an oil formulation) for 30 days (N=10 neonates)	not provided	Primary/secondary outcome(s): gastric electrical activity (propagation), gastric emptying, weight gain, adverse events
Manzoni et al. 2013	multi center RCT N=229 neonates	Inclusion: gestational age <32 weeks + 6 days, born within the study period<br><br>Exclusion: lack of parental consent, admission >48 hours of life, death <72 hours of life and ophthalmic disease present at moment of randomization	oral placebo supplementation, 0.5 mL glucose 5% (N=116 neonates)	oral carotenoids supplementation, 0.5 ml containing 0.14 mg lutein and 0.0006 mg of zeacanthin, single daily oral dose starting from the first 48 hours of life until week 36 of corrected gestational age (N=113 neonates)	Yes, power calculation based on pretrial retinopathy of prematurity incidence of 18%, estimated 66% relative difference in retinopathy of prematurity incidence by carotenoids supplementation, power 80%; α 0.05; required sample size 114	Primary outcome(s): incidence threshold retinopathy of prematurity, incidence bronchopulmonary dysplasia, incidence NEC and incidence NEC stage ≥2<br><br>Secondary outcome(s): incidence retinopathy of prematurity (all stage), intestinal perforation, late-onset sepsis, mortality prior to discharge, severe (grade 3 or 4) intraventricular hemorrhage, need for

						transfusion, liver failure
Manzoni et al. 2014	multi center RCT N=472	Inclusion: VLBW neonates younger than 3 days at tertiary care units in Italy and New Zealand<br><br>Exclusion: lack of parental consent, ongoing antifungal prophylaxis, early onset	placebo (2 mL of a 5% glucose solution from day 3 once daily dissolved in milk for 6 (birth weight <1000 g) or 4 (birth weight 1001-1500g) weeks or until discharge (N=168 neonates)	bovine lactoferrin, 100 mg/day dissolved in milk, orally once daily from day 3 for 6 (birth weight <1000 g) or 4 (birth weight 1001-1500g) weeks or until discharge (N=153 neonates)<br><br>bovine lactoferrin, 100 mg/day dissolved in milk, + Lactobacillus rhamnosus GG, $6*10^9$ CFU / day dissolved in milk, orally once daily from day 3 for 6 (birth weight <1000 g) or 4 (birth weight 1001-1500g) weeks or until discharge (N=151 neonates)	Yes, power calculation based on pretrial NEC incidence of 7%, estimated 66% relative difference in NEC incidence by supplementation, power 80%; α 0.05; required sample size 238<br><br>NB: due to lower incidence rate of NEC (5.4% instead of 7%) power fell to 54% for the bovine lactoferrin supplementation group and 97% for lactoferrin + Lactobacillus rhamnosus GG group	Primary outcome(s): NEC stage ≥2 and NEC or death<br><br>Secondary outcome(s): overall mortality (not attributable to NEC)
Mihatsch et al. 2006	single center RCT N=20 neonates	Inclusion: healthy, stable, preterm infants with birthweight <1500 gram and full enteral feeding with preterm	standard preterm formula supplemented with placebo (1 g/dL, made from sachets	standard preterm formula supplemented with GOS FOS (1g/dL, GOS FOS 1:1, made from sachets with 0.9g	no power calculation performed (pilot study)	Primary outcome(s): stool viscosity, gastrointestinal transit time

		infant formula because breastmilk was unavailable<br><br>Exclusion: lack of parental consent, anomalies that may interfere with feeding tolerance (cardiac defects, pulmonary diseases such as bronchopulmonary dysplasia, gastrointestinal diseases or chromosomal abnormalities)	with 1.8g maltodextrin) (N=10 neonates)	GOS FOS and 0.9g maltodextrin) (N=10 neonates)		Secondary outcome(s): stool frequency and quality, stool pH, feeding volume, weight gain
Omar et al. 2020	single center, double-blind placebo controlled pilot study, N=120 neonates	Inclusion gestational age ≤ 32 weeks at the NICU of a tertiary care hospital<br><br>Exclusion: lack of parental consent, receiving chest compression or any medication during resuscitation, genetic syndromes or inborn errors of metabolism, major congenital defects, previous administration of parenteral growth	single daily dose of distilled water mixed with preterm formula (N=60 neonates)	enteral recombinant EPO, 0.88 IU/kg/day at concentration of 100 IU/mL in distilled water mixed with preterm formula, single enteral daily dose, starting at the day of enteral feeding until enteral intake of 150/ml/kg or after a maximum of 10 days (N=60 neonates)	Yes, a sample size of 30 patients per group was deemed sufficient for a pilot study with power of 80% and α 0.05	Primary outcome(s): feeding tolerance (including day of successful start of enteral feeding).<br><br>Secondary outcome(s): time to establish one-half, two-thirds and full enteral feedings, number of episodes of feeding intolerance, time to regain birth weight, incidence of NEC (stage ≥2), adverse effects

		factors or previous treatment with intravenous immunoglobulins				
Serce Pehlevan et al. doi: 10.5546/aap.2020.eng.e8 2020	single center RCT N=50 neonates	Inclusion: gestational age ≤32 weeks, admitted to the neonatology unit of the Zeynep Kamil Maternity and Children's Training and Research Hospital,Istanbul, Turkey, between July and September 2013<br><br>Exclusion: congenital anomalies, born to mothers with premature rupture of membranes or chorioamnionitis<br><br>Excluded after randomization: mechanical ventilation for >7 days, culture proven sepsis at time of blood sampling, NEC, surgery, infants that died <30 days of life	placebo (distilled water 1 mL per dose, every 12 hours), added to breast milk or formula	mixture containing per sachet 8.2*10^8 *Lactobacillus rhamnosus* KCTC 12202BP, 4.1 *10^8 *Lactobacillus plantarum* KCTC 10782BP, *Lactobacillus casei* KCTC 12398BP, *Bifidobacterium lactis* KCTC 11904BP, 383 mg FOS, 100 mg GOS and 2 mg bovine lactoferrin, ½ sachet every 12 hours added to breast milk or formula	Yes, with an estimated effect size of 0.75 SD, a power of 80% and α 0.05, 24 patients were needed per group	Primary/secondary outcome(s): serum cytokine levels (IL5, IL10, IL17a, IFNϒ)
Serce Pehlevan et al. doi:	single center RCT N=208 neonates	Inclusion: gestational age ≤32 weeks, birth weight ≤1500 g, admitted to the	placebo (distilled water 1 mL per dose, every 12 hours), added to	mixture containing per sachet 8.2*10^8 *Lactobacillus rhamnosus* KCTC 12202BP, 4.1 *10^8	Yes, power calculation based on pretrial NEC or late onset sepsis	Primary outcome(s): NEC stage ≥2 or culture proven late onset sepsis,

10.3345/cep.2019.00381<br>2020		neonatology unit of the Zeynep Kamil Maternity and Children's Training and Research Hospital,Istanbul, Turkey, between February 2012 and September 2013, survived to start of enteral feeding<br><br>Exclusion: severe asphyxia (stage III), major congenital anomalies, fasted ≥3 weeks, death within first 14 postnatal days	breast milk or formula	*Lactobacillus plantarum* KCTC 10782BP, *Lactobacillus casei* KCTC 12398BP, *Bifidobacterium lactis* KCTC 11904BP, 383 mg FOS, 100 mg GOS and 2 mg bovine lactoferrin, ½ sachet every 12 hours added to breast milk or formula	incidence of 31% and death or NEC incidence of 35%, estimated 50% difference in NEC/late onset sepsis or NEC/death incidence by supplementation, power 80%; α 0.05; required sample size of 104 for NEC or late onset sepsis and sample size of 92 for NEC or death	NEC stage ≥2 or death<br><br>Secondary outcome(s): time to reach 100 ml/kg/day of enteral feeding, oxygen dependency at 36 weeks, mortality before hospital discharge and duration of hospitalization
Sevastiadou et al.<br>2011	single center RCT<br>N=101 neonates	Inclusion: formula fed, prematurely born infants admitted to the NICU of 'Alexandra' Regional General Hospital, Athens, Greece, between January 2007 and December 2008 with a GA <34 weeks and birth weight <2000 g<br><br>Exclusion: lack of parental consent, major congenital or chromosomal	isocaloric glucose-polymer pounder with same color and smell as intervention pounder<br>(N=50 neonates)	enteral L-glutamin pounder supplementation separate from formula, 3 times a day between 3 and 30 days of life, 0.3 g/kg/day diluted in water as a 10% solution<br>(N=51 neonates)	not provided	Primary/secondary outcome(s): intestinal permeability, NEC incidence, sepsis

		anomalies, severe hypotension, severe perinatal distress (pH <7 or hypoxia with bradycardia > 2h), abdominal distention, signs of early NEC, receiving breast milk				
Stratiki et al. 2007	RCT<br>N=75 neonates	Inclusion:<br>gestational age between 27 and 37 gestational age, stable state, formula fed and not suffering from major deformities (congenital heart defects, bowel atresia)	formula without supplemented *Bifidobacterium lactis*	*Bifidobacterium lactis* in formula feeding, 2*107 CFU/g milk powder, administered according to neonatal unit feeding protocol	Yes, based on data on lactulose / mannitol ratio from an earlier study, a sample size of 30 infants was considered adequate to detect a significant difference of lactulose / mannitol ratio following supplementation	Primary outcome(s): intestinal permeability<br><br>Secondary outcome(s): somatic growth, tolerance, rates of sepsis and NEC
Sun et al. 2020	multi center RCT<br>N=262 neonates	Inclusion: gestational age <28 weeks and <96 hours of age<br><br>Exclusion: lack of parental consent, genetic metabolic disease, congenital major abnormalities, congenital TORCH infections with overt signs at birth, terminal	placebo solution with soybean oil with the same aspect as vitamin A solution<br>(N=130 neonates)	Vitamin A, 1500 IU/day, solution was added to enteral feeds when minimal enteral feeding started until 28 days or discharge<br>(N=132 neonates)	Yes, power calculation based on estimated 30% relative difference in retinopathy of prematurity incidence by supplementation, power 80%; α 0.05; required sample size 254	Primary outcome(s): composite of mortality or type 1 retinopathy of prematurity, bronchopulmonary dysplasia, serum vitamin A levels, sings of vitamin A toxicity, vomiting and intracranial pressure

		stage illness (pH <7.0 or hypoxia with bradycardia >2 hours)				Secondary outcome(s): mortality, retinopathy of prematurity, sepsis, NEC stage ≥2, severe intraventricular hemorrhage (grade ≥3), periventricular leukomalacia
Wardle et al. 2001	single center RCT N=154 neonates	Inclusion: <1000g birth weight and parental consent within the first 24 hours<br><br>Exclusion: lack of parental consent, major life threatening congenital abnormality	daily dose of equal volume of an inert placebo solution (looking identical as the intervention) orally as a bolus through orogastric tube from postnatal day 1 until day 28 (N= 77 neonates)	vitamin A, daily dose of 5000 IU/kg (3000 µg/kg) orally as a bolus through orogastric tube from postnatal day 1 until day 28 (N= 77 neonates)	Yes, power calculation based on prior incidence of chronic lung disease death of 90% and of chronic lung disease in survivors of 83%, estimated risk reduction to 70% (chronic lung disease and death) and 50% (chronic lung disease), power 80%; α 0.05; required sample size 158	Primary outcome(s): requirement for supplementary oxygen at 28 days<br><br>Secondary outcome(s): death before discharge, supplementary oxygen requirement at 36 weeks, retinopathy of prematurity requiring treatment, intraventricular hemorrhage with parenchymal involvement, patent ductus arteriosus requiring treatment, NEC requiring surgery and number of episodes of sepsis, adverse events

Westerbeek et al. 2011	single center RCT N=113 neonates	Inclusion: preterm infants <32 weeks GA and/or birthweight <1500 g admitted to the NICU of the VU University Medical Center, Amsterdam.<br><br>Exclusion: >34 weeks GA, major congenital or chromosomal anomalies, imminent death, transfer to another hospital <48h after birth	placebo powder (maltodextrin) in increasing doses between d3 and d30 to a maximum of 1.5 g/kg added to breastmilk or preterm formula (without oligosaccharides) (N= 58 neonates)	72% SC-GOS, 8% LC-FOS, 20% AOS in increasing doses between d3 and d30 to a maximum of 1.5 g/kg added to breastmilk or preterm formula (without oligosaccharides) (N= 55 neonates)	Yes, the sample size of 113 was based on sample size calculation for the primary outcome of the main trial (serious infectious morbidity)	Primary/secondary outcome(s): fecal calprotectin, fecal IL8

RCT, randomized controlled trial; GA, gestational age; NEC; necrotizing enterocolitis; SC-GOS, short-chain galacto-oligosaccharides; LC-FOS, long-chain fructo-oligosaccharides; DHA, docosahexaenoic acid; EPA: eicosapentaenoic acid; EPO, erythropoietin; G-CSF, granulocyte colony-stimulating factor; AOS, acidic oligosaccharides

Supplementary table S7. Overview included systematic reviews and meta-analysis on enteral feeding interventions for prevention of NEC in human infants

Author and year	Type of study and sample size	Study selection criteria	Control	Intervention	Primary / secondary outcome measures	Evidence level and risk of bias according to authors meta-analysis
Van den Akker et al. 2018	systematic review and network meta-analysis<br><br>N for NEC outcome = 43 studies and 10.651 neonates	RCTs reporting on only preterm infants or subgroup with preterm infants (<37 weeks GA), comparing probiotic treatment with placebo or no treatment or comparing different probiotic interventions, both single- or multiple strains included, available in English and fully published	placebo, usual care or head-to-head with different probiotic regime	probiotics single- or multiple-strain	Primary outcomes: well-described outcome reports of NEC, blood-culture proven late onset sepsis, postnatal age at reaching full enteral feeding, in-hospital mortality<br><br>Secondary outcome: -	studies in general underpowered for NEC incidence as outcome measure, most strains only studied in a few trials, limited evidence for the most preterm infants
Ananthan et al. 2018	systematic review and meta-analysis<br><br>N for NEC outcome (enteral administration) = 2 studies and 110 neonates	RCTs comparing recombinant EPO with placebo or standard treatment without recombinant EPO supplementation	placebo or standard treatment without EPO	recombinant EPO	Primary outcomes: incidence of NEC stage ≥2, any stage NEC<br><br>Secondary outcomes: feeding intolerance, time to reach full feedings, late onset sepsis, bronchopulmonary dysplasia, mortality	the included trials for enteral administration were at risk for selection bias, reporting bias and other bias
Chi et al. 2019	systematic review and meta-analysis	RCTs comparing prebiotics (SC-GOS, LC-FOS, pAOS, oligosaccharides,	placebo	prebiotics (SC-GOS, LC-FOS, pAOS, oligosaccharides,	Primary outcomes: incidence of sepsis, NEC and mortality<br><br>Secondary outcomes: length of	5 out of 6 included studies were judged to be of high quality, 1

	N for NEC outcome = 6 studies and 737 neonates	fructans, inulin or oligofructose) in low birth weight (<2500 g) or preterm(<37 weeks GA) infants with placebo, published in peer-reviewed journals from January 2020 until June 2018		fructans, inulin or oligofructose)	hospital stay, feeding intolerance, stool frequency	study was of moderate quality
Foster et al. 2016	systematic review and meta-analysis<br><br>N for NEC outcome = 3 studies and 2095 neonates	RCTs and quasi-randomized controlled trials studying the use of oral immunoglobulins as prophylaxis against NEC in preterm (<37 weeks GA) and/or low birth weight (<2500 g) neonates	placebo or no treatment	oral immunoglobulins	Primary outcome: diagnosis of definite NEC during study period<br><br>Secondary outcomes: suspected NEC during study period, surgery for NEC during study period, NEC related death, length of hospital stay, hospital readmission within the first year of life, days receiving total parenteral nutrition, growth and development in childhood, parental emotional and financial costs, adverse effects of treatment	low to very low quality of evidence; incomplete outcome data, high rate of non-compliance, unclear allocation concealment and imprecision
Moe-Byrne et al. 2016	systematic review and meta-analysis<br><br>N for NEC outcome = 7 studies and 1172 neonates	RCTs and quasi-randomized controlled trials of glutamine supplementation in preterm neonates at any time from birth to discharge from hospital	no supplementation	enteral (or parenteral) glutamine supplementation from any time from birth until hospital discharge	Primary outcomes: death prior to hospital discharge, neurodevelopment<br><br>Secondary outcomes: invasive infection during hospital admission, NEC during hospital admission	moderate quality of evidence; unexplained heterogeneity and funnel plot asymmetry

Morgan et al. 2020	systematic review and network meta-analysis<br><br>N for NEC outcome = 56 studies and 12738 neonates	RCTs with single- or multiple-strain probiotic (living bacteria) interventions for prevention of mortality or morbidity in preterm (<37 weeks GA) and/or low birth weight (<2500 gram) infants	placebo, formula, parenteral nutrition or no treatment	probiotics (living bacteria) single- or multiple-strain	Primary outcomes: all-cause mortality, severe NEC (stage ≥2), culture proven sepsis, hospitalization, time to reach full enteral feeds<br><br>Secondary outcome: -	low to high certainty of evidence; data with low and moderate to high certainty of evidence are presented separately
Pammi et al. 2020	systematic review and meta-analysis<br><br>N for NEC outcome = 7 studies and 4874 neonates	RCTs evaluating the effect of enteral lactoferrin administration at any dose or duration to prevent NEC (and sepsis) in preterm neonates	placebo or no intervention	enteral lactoferrin at any dose or duration	Primary outcomes: confirmed or suspected sepsis during hospital stay, NEC stage ≥2, all-cause mortality during hospital stay<br><br>Secondary outcomes: neurological outcome at two years of age or alter, chronic lung disease in survivors, adverse outcomes, periventricular leukomalacia, duration of assisted ventilation through an endotracheal tube, length of hospital stay, posthoc analyses of bacterial infection, fungal infection, threshold retinopathy of prematurity and urinary tract infection	low certainty of evidence for NEC outcome; confidence in the effect estimate is limited, true effect may be substantially different from the estimate of the effect, risk of bias in the included trials and imprecision
Shah et al. 2017	systematic review and meta-analysis	RCTs and quasi-randomized controlled trials of arginine	placebo or no treatment	enteral or parenteral arginine	Primary outcome: NEC any stage and specific stages	moderate certainty of evidence; future research likely will

	N for NEC outcome = 3 studies and 285 neonates	supplementation, administered orally or parenterally for at least seven days, in addition to intake with regular enteral/parenteral feeding		supplementation for at least 7 days	Secondary outcomes: death before discharge, death attributed to NEC, surgery for NEC, duration of total parenteral nutrition administration, plasma concentrations of arginine and glutamine, side effects of arginine supplementation	have an important impact on confidence in the estimate of effect and may change this estimate
Zhang et al. 2014	systematic review and meta-analysis<br><br>N for NEC outcome = 5 studies and 900 neonates	observational studies or RCTs published until May 2013 that included infants <29 weeks GA that evaluated the relationship between n-3 long chain PUFA and major adverse neonatal outcomes	standard interventions, placebo or any other control levels of n-3 long chain PUFA exposure	any n-3 long chain PUFA exposure to the infant or any n-3 long chain PUFA supplementation directly to the infant or through the mother	Primary outcome: bronchopulmonary dysplasia free survival at 36 weeks postmenstrual age<br><br>Secondary outcomes: death, duration of ventilation or oxygen support, length of hospitalization, occurrence of intraventricular hemorrhage, periventricular leukomalacia, NEC, infections, retinopathy of prematurity or hemodynamic significant patent ductus arteriosus	no separate quality analysis reported for the included studies

RCT, randomized controlled trial; GA, gestational age; NEC; necrotizing enterocolitis; EPO, erythropoietin; SC-GOS, short chain galacto-oligosaccharides; LC-FOS, long chain fructo-oligosaccharides; pAOS: pectin-derived acidic oligosaccharides; PUFA, polyunsaturated fatty acids

Supplementary table S8. Risk of bias assessment of the included experimental animal studies (SYRCLE's risk of bias tool)

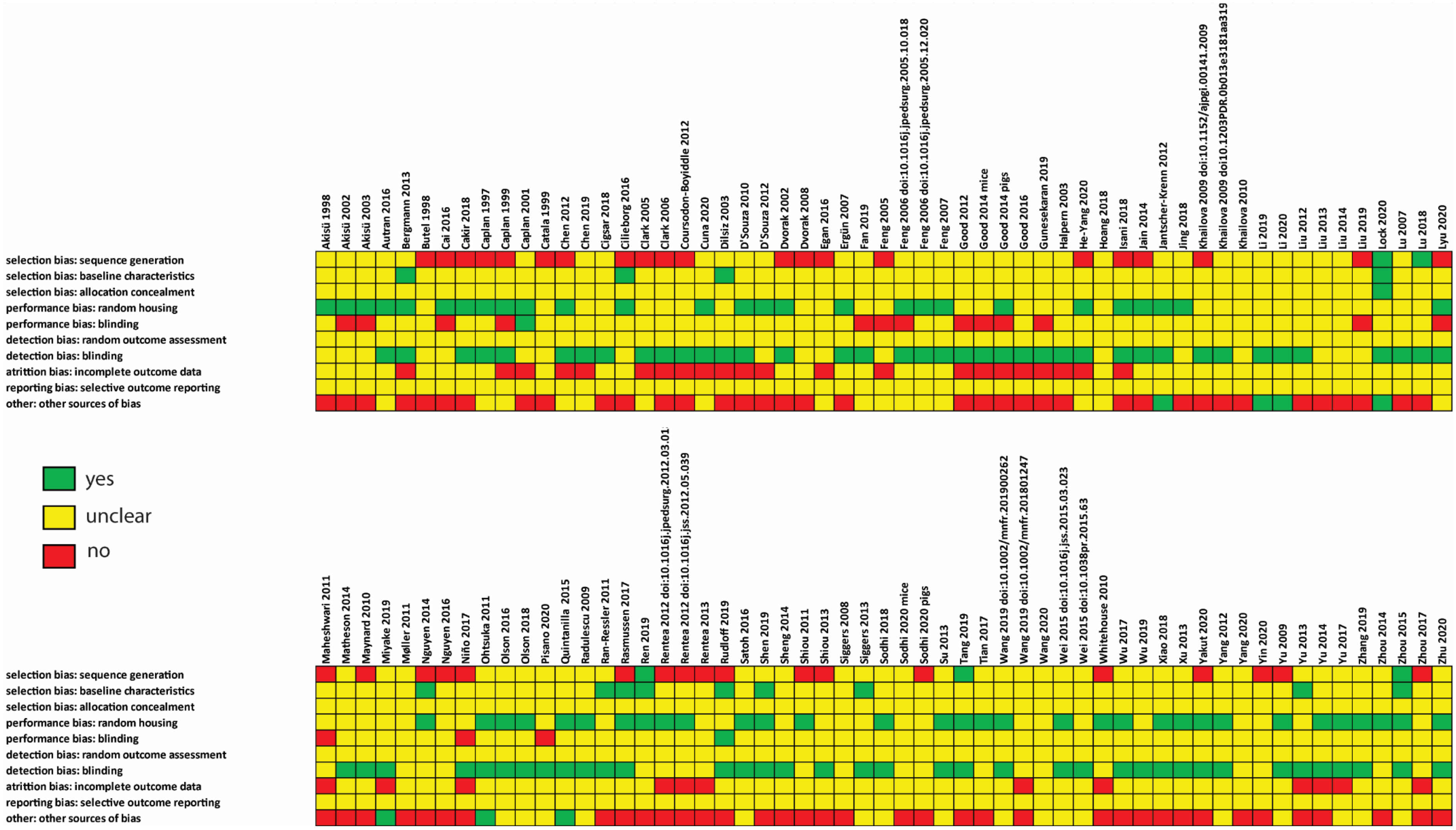

Supplementary table S9. Risk of bias assessment of the included RCTs (Jadad scoring system)

	Akin 2014	Collins 2017	El-Ganzoury 2014	Hosseini 2018	Indrio 2009	Manzoni 2013	Manzoni 2014	Mihatsch 2006	Omar 2020	Serce Pehlevan 2020 doi: 10.5546/aap.2020.eng.e8	Serce Pehlevan 2020 doi: 10.3345/cep.2019.00381	Sevastiadou 2011	Stratiki 2007	Sun 2020	Wardle 2001	Westerbeek 2011
1) Was the study described as randomized	2	2	2	1	1	2	2	2	2	2	2	1	2	1	2	2
2) Was the study described as double-blind	2	2	2	0	2	2	2	2	2	2	2	2	2	2	2	2
3) Was there a description of withdrawels and dropouts	1	1	1	1	0	1	1	1	1	1	1	1	1	1	0	1
Total score	5	5	5	2	3	5	5	5	5	5	5	4	5	4	4	5

Supplementary table S10. Risk of bias assessment of the included meta-analyses (AMSTAR measurement tool)

	Akker, van den 2018	Ananthan 2018	Chi 2018	Foster 2016	Moe-Byrne 2016	Morgan 2020	Pammi 2020	Shah 2017	Zhang 2014
1) Was an 'a priori' design provided?	yes	can't answer	yes	yes	yes	yes	yes	yes	yes
2) Was there duplicate study selection and data extraction?	yes	yes	yes	yes	yes	yes	yes	yes	yes
3) Was a comprehensive literature search performed?	no	yes	yes	yes	yes	yes	yes	yes	yes
4) Was the status of publication (i.e. grey literature) used as an inclusion criterion?	yes	yes	can't answer	yes	yes	yes	yes	yes	yes
5) Was a list of studies (included and excluded) provided?	no	no	no	yes	yes	can't answer	yes	no	no
6) Where the characteristics of the included studies provided?	yes	yes	yes	yes	yes	yes	yes	yes	yes
7) Was the scientific quality of the included studies assessed and documented?	yes	yes	yes	yes	yes	yes	yes	yes	yes
8) Was the scientific quality of the included studies used appropriately in formulating conclusions?	yes	yes	can't answer	yes	yes	yes	yes	yes	can't answer
9) Where there methods used to combine the findings of studies appropriate?	yes	yes	yes	yes	yes	yes	yes	yes	yes
10) Was the likelihood of publication bias assessed?	yes	yes	yes	yes	yes	yes	yes	yes	yes
10) Was the conflict of interest stated?	no	no	no	no	no	yes	no	no	no

Supplementary table S11. GRADE approach scoring of clinical studies

	limitations in study design or execution (risk of bias)	inconsistency of results	indirectness of evidence	imprecision	Publication bias	Overall certainty of evidence (GRADE)
n-3 PUFA (NEC incidence all neonates)	-1	0	0	-1	0	Low
n-3 PUFA (NEC incidence neonates ≤32 weeks)	-1	0	0	-1	0	Low
DHA (NEC incidence)	0	0	0	-2	0	Low
prebiotics (NEC incidence)	0	-1	0	-1	0	Low
lactoferrin (incidence stage II or III NEC)	-1	0	0	-1	0	Low
arginine (NEC incidence)	0	0	0	-1	0	Moderate
arginine (death due to NEC)	0	0	0	-2	0	Low
glutamine (NEC incidence)	0	0	0	-1	-1	Low
immunoglobulins, (NEC incidence)	-2	0	0	0	0	Low
immunoglobulins, (NEC surgery)	-2	0	0	-1	0	Very low
immunoglobulins (death due to NEC)	-2	0	0	-1	0	Very low
EPO (incidence stage II or III NEC)	0	-1	0	-2	0	Very low
EPO (NEC incidence)	-1	-1	0	-2	0	Very low
EPO + G-CSF (NEC incidence)	0	0	0	-2	0	Low
G-CSF (NEC incidence)	0	0	0	-2	0	Low
G-CSF (in artificial amniotic fluid) (NEC incidence)	-1	0	0	-2	0	Very low
vitamin A (NEC incidence)	-1	0	0	-1	0	Low
probiotics Lactobacillus spp. and Bifidobacterium spp. (incidence stage II or III NEC)	0	0	-1	0	0	Moderate
probiotics Bifodobacterium animalis subsp. Lactis (incidence stage II or III NEC)	0	0	0	-1	0	Moderate
probiotics Lactobacillus reuteri (incidence stage II or III NEC)	0	0	-1	-1	0	Low
probiotics Lactobacillus rhamnosus (incidence stage II or III NEC)	0	0	0	-1	0	Moderate
probiotics combination of Lactobacillus ssp., Bifidobacterium spp. and Enterococcus ssp. (incidence stage II or III NEC)	-2	0	0	0	0	Low
probiotics combination of Bacillus spp. and Enterococcus spp. (incidence stage II or III NEC)	0	0	0	-1	0	Moderate
probiotics Bifidobacterium lactis Bb-12 or B-94 (NEC incidence)	0	0	0	-1	0	Moderate
probiotics Lactobacillus reuteri ATCC55730 or DSM17938 (NEC incidence)	0	0	-1	-1	0	Low
probiotics Lactobacillus rhamnosus GG (NEC incidence)	0	0	-1	-1	0	Low
probiotics combination of Bifidobacterium bifidum, Bifidobacterium infantis, Bifidobacteirum longum and Lactobacillus acidophilus (NEC incidence)	0	0	0	-2	0	Low
probiotics combination of Bifidobacterium infantis Bb-02, Bifidobacterium lactis Bb-12 and Streptococcus thermophilus TH-4 (NEC incidence)	0	-1	0	-1	0	Low
probiotics Bifidobacterium longum 35624 and Lacobacillus rhamnosus GG (NEC incidence)	0	0	0	-2	0	Low
carotenoids (incidence stage II or III NEC)	0	0	0	-2	0	Low
mixture of probiotics, prebiotics and lactoferrin (NEC incidence)	0	0	0	-2	0	Low
mixture of probiotics, prebiotics and lactoferrin (incidence stage II or III NEC)	0	0	0	-2	0	Low
